# Characteristics, Physiopathology and Management of Dyslipidemias in Pregnancy: A Narrative Review

**DOI:** 10.3390/nu16172927

**Published:** 2024-09-01

**Authors:** Elena Formisano, Elisa Proietti, Giuseppina Perrone, Valentina Demarco, Paola Galoppi, Claudia Stefanutti, Livia Pisciotta

**Affiliations:** 1 Department of Internal Medicine, University of Genoa, 16132 Genoa, Italy; elena.formisano@hsanmartino.it (E.F.); elisa.proietti@edu.unige.it (E.P.); 2 Dietetics and Clinical Nutrition Unit, IRCCS Policlinic Hospital San Martino, 16132 Genoa, Italy; 3 Department of Maternal and Child Health and Urological Sciences, Umberto I Hospital, “Sapienza” University of Rome, 00161 Rome, Italy; giuseppina.perrone@gmail.com (G.P.); paola.galoppi@uniroma1.it (P.G.); 4 Department of Molecular Medicine, Extracorporeal Therapeutic Techniques Unit, Lipid Clinic and Atherosclerosis Prevention Centre, Regional Centre for Rare Diseases, Immunohematology and Transfusion Medicine, Umberto I Hospital, “Sapienza” University of Rome, 00161 Rome, Italy; claudia.stefanutti@uniroma1.it

**Keywords:** pregnancy, dyslipidemia, familial hypercholesterolemia

## Abstract

Dyslipidemia is a significant risk factor for atherosclerotic cardiovascular disease (ASCVD). During pregnancy, physiological changes elevate cholesterol and triglyceride levels to support fetal development, which can exacerbate pre-existing conditions and lead to complications such as pre-eclampsia, gestational diabetes, and increased ASCVD risk for both mother and child. Effective management strategies are necessary, especially for pregnant women with inherited forms of dyslipidemia (i.e., familial hypertriglyceridemia, hyperchylomicronemia), where personalized dietary adjustments are crucial for successful pregnancy outcomes. Pharmacological interventions and lipoprotein apheresis may be necessary for severe cases, though their use is often limited by factors such as cost, availability, and potential fetal risks. Despite the promise of advanced therapies, their widespread application remains constrained by limited studies and high costs. Thus, a personalized, multidisciplinary approach is essential for optimizing outcomes. This review provides a comprehensive overview of current strategies and evidence-based practices for managing dyslipidemia during pregnancy, emphasizing the balance of maternal and fetal health. Additionally, it discusses the physiological changes in lipid metabolism during pregnancy and their implications, particularly for women with inherited forms of dyslipidemia.

## 1. Introduction

Dyslipidemia is characterized by abnormal lipid levels in the blood and represents a significant risk factor for atherosclerotic cardiovascular disease (ASCVD), which remains a leading cause of morbidity and mortality globally [1,2]. The etiology of dyslipidemia is multifaceted, involving genetic predispositions, lifestyle factors and other risk factors, including obesity, diabetes, and hypertension [3,4,5]. Variations in the lipid profile can also be observed across different physiological states, from childhood and adolescence to adulthood throughout the aging process (i.e., the menopausal period for women), influenced by hormonal, genetical and environmental factors [6,7,8]. In this regard, pregnancy represents a special physiological state during which the lipid profile undergoes significant increases to support fetal development, providing the fetus with essential lipids. However, this can also pose challenges for women with pre-existing genetic dyslipidemia, or gestational lipid abnormalities [9]. The management of dyslipidemia in pregnancy focuses on balancing the nutritional needs of the mother and fetus while minimizing the risks to both [10].

Evidence shows that elevated supraphysiological levels of total cholesterol (TC), low-density lipoprotein cholesterol (LDL-C), triglycerides (TG), and reduced high-density lipoprotein cholesterol (HDL-C), could pose significant risks, including pre-eclampsia, gestational diabetes, and an increased ASCVD risk later in life for both mother and offspring [11,12,13]. Thus, it is pivotal to better clarify the management of dyslipidemia during this physiological state, considering that addressing dyslipidemia in pregnancy is a delicate balancing act, necessitating interventions that are safe, effective, and devoid of adverse effects on fetal development.

The 2019 guidelines from the European Society of Cardiology (ESC) and the European Atherosclerosis Society (EAS) provide some nuanced insights into managing dyslipidemia in specific situations, including pregnancy, but do not fully delve into the topic, due to the limited evidence available in this field [14]. The Mediterranean diet (MD), a first-line strategy for treating dyslipidemia, is recognized as a healthy eating pattern that meets the increased nutritional needs associated with the unique physiological state of pregnancy [15,16,17]. However, when dietary and lifestyle modifications are insufficient to achieve target lipid levels, pharmacological interventions may be considered [14,18,19]. Nevertheless, the use of lipid-lowering medications during pregnancy requires careful consideration of the benefits and potential risks to both the mother and the fetus [20].

This narrative review aims to explore the relationship between dyslipidemia and pregnancy, analyzing the changes in lipid profile during pregnancy and the possible adverse outcomes that could be related to the presence of dyslipidemia, aiming to provide a comprehensive overview of current strategies for managing dyslipidemia in the context of pregnancy, guided by the latest evidence-based practices.

## 2. Lipid Physiology in Pregnancy

**Figure 1:**
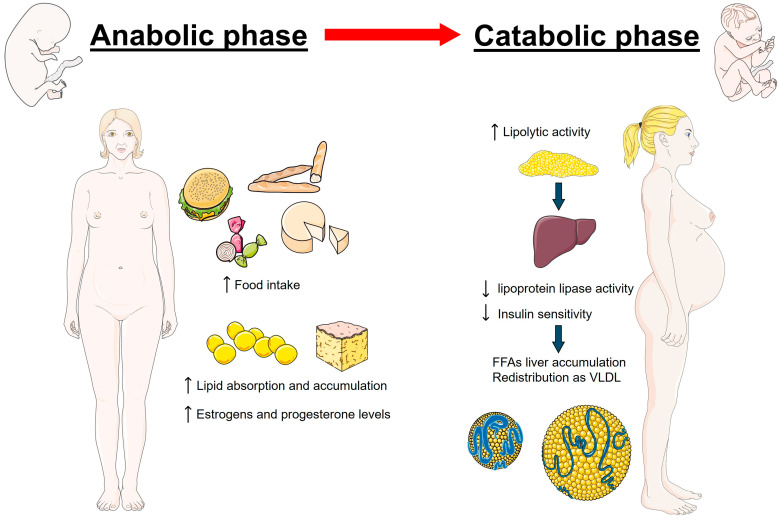
Metabolic changes during pregnancy initially involve an anabolic state, marked by increased food intake and lipid accumulation. As pregnancy progresses, it shifts to a catabolic state, characterized by enhanced lipolysis and elevated maternal lipid levels in the bloodstream. These adaptations are driven by hormonal changes, particularly the rise in estrogen and progesterone.

Pregnancy is characterized by great physiological changes, among which alterations in lipid metabolism are essential for supporting fetal development and ensuring successful pregnancy outcomes [21,22]. The first half of pregnancy is characterized by an anabolic state. During this period, there is a remarkable increase in appetite and food intake (hyperphagia) alongside enhanced lipid synthesis and accumulation, serving as a reserve for the energy demands that will escalate in the later stages of pregnancy [22,23,24]. This physiological adaptation is driven by hormonal changes, particularly increases in estrogen and progesterone, which stimulate the synthesis of TG and cholesterol in the liver [22,25]. As pregnancy advances into its latter stages, the metabolic state of the body transitions towards catabolism, highlighted by an enhanced lipolytic activity that enhances the breakdown of stored fats into free fatty acids (FFAs) and glycerol [26,27]. This period is distinguished by reduced insulin sensitivity, a strategic physiological adjustment aimed at maintaining a consistent glucose supply to the fetus, despite leading to increased levels of maternal lipids [26]. The decrease in lipoprotein lipase activity, in conjunction with insulin resistance, hampers the assimilation of TG from the plasma into adipose tissue, resulting in their accumulation in the bloodstream [28]. Given the limited capacity for the placental transfer of lipolysis products such as FFAs and glycerol [29], these substances are activated and re-esterified in the maternal liver, then redistributed into circulation as very low-density lipoproteins (VLDL) [30]. Consequently, there is a significant rise in circulating levels of TG (+50–100%), TC (+30–50%), and LDL-C (+30–50%) [9,31,32]. It is important to note that normal lipid values during pregnancy should be considered higher than in the general female population according to different ethnic groups. Specifically, during early pregnancy, the recommended reference values for serum lipids should be TC less than 218 mg/dL, TG less than 173 mg/dL, HDL-C greater than 47 mg/dL, and LDL-C less than 126 mg/dL. In mid-pregnancy, the recommended lipid levels should be TC less than 290 mg/dL, TG less than 315 mg/dL, HDL-C greater than 54 mg/dL, and LDL-C less than 187 mg/dL [33]. This lipid profile adaptation not only supports the energy-intensive process of lactation but also supplies essential fatty acids and cholesterol necessary for fetal nervous system development and the construction of cellular membranes [34,35]. Figure 1 illustrates the metabolic changes during pregnancy.

Furthermore, the condition of hypertriglyceridemia can ensure glucose provision to the fetus during fasting states, as TG are utilized by the mother to synthesize ketone bodies [27]. This process offers an alternative energy source, maintaining fetal glucose levels even when maternal glucose intake is limited. However, the great escalation in TG levels, potentially tripling by the third trimester, represents a critical adaptation, as it could contribute to increasing the risk of the development of lipid-related complications [36].

Nevertheless, it is important to consider that lipid measurements evolve during pregnancy, and there is uncertainty about the extent to which these changes are physiological or pathological. Additionally, normal values can vary with ethnicity or gestational age at the time of sampling [37,38].

Special attention must be given to pregnant women with familial hypercholesterolemia (FH), an autosomal dominant condition characterized by elevated LDL-C levels, normal TG levels, cholesterol deposits in tendons and skin (xanthomas), and an increased risk of premature ASCVD [39,40,41]. Managing lipid levels in these patients is crucial due to their significantly elevated baseline lipid profiles compared to the general population. Indeed, pregnant women with FH are at an increased risk of developing cardiovascular events, further exacerbating the already heightened risk of cardiovascular complications associated with pregnancy due to more pronounced alterations in their lipid profiles [42]. However, evidence suggests that despite their higher baseline lipid levels, the relative increase in lipid fractions during pregnancy in women suffering from FH is similar to that of healthy pregnant women [43]

## 3. Hyperlipidemia and Possible Adverse Maternal and Neonatal Outcomes

Evidence suggests that dyslipidemia during pregnancy is associated with the development of different diseases, primarily of metabolic interest, which could not only affect pregnancy outcomes but also have long-term implications for cardiovascular health [44].

Pregnant women with dyslipidemia, especially those with elevated TG levels early in pregnancy, are at an increased risk of developing gestational diabetes, as both conditions share underlying mechanisms related to insulin resistance [45]. A meta-analysis by Ryckman et al., involving 60 studies with 4168 women diagnosed with gestational diabetes mellitus (GDM) and 9718 controls, demonstrated significant differences in lipid profiles. Women with GDM had significant higher TG levels and lower HDL-C levels compared to those without GDM. However, there were no significant differences in TC and LDL-C levels between the groups [46]. Additionally, another meta-analysis by Rahnemaei et al. found that the largest difference between the GDM and control groups was in TG levels, concluding that this lipid fraction had the strongest impact on GDM [47]. Likewise, overweight and obesity during pregnancy exacerbate the risk of both dyslipidemia and GDM [47,48]. Excessive body weight can further impair insulin sensitivity, creating a more pronounced predisposition towards insulin resistance [49]. This condition is a key factor in the development of gestational diabetes, making overweight pregnant women particularly susceptible. Furthermore, obesity is closely linked with alterations in lipid metabolism, contributing to an adverse lipid profile characterized by elevated TG and reduced HDL-C levels [50,51]. In a prospective observational study, it was found that GDM and dyslipidemia are significantly associated, especially in the context of maternal obesity. Among 275 women, those with GDM had higher TG and lower HDL-C levels compared to those without GDM. This association was particularly pronounced in women with obesity, suggesting that the link between GDM and dyslipidemia is mediated by maternal obesity [48]. This latter condition is frequently associated with a higher risk of developing pre-eclampsia, although the underlying mechanism remains not fully understood, indicating a potential link with alterations in lipid profiles [52]. Evidence suggests that disruptions in placental development or functionality might underpin pre-eclampsia, leading to a compromised exchange of nutrients and oxygen between mother and child, as well as oxidative stress accompanied by the release of antiangiogenic factors into the blood that could damage the maternal vascular endothelium and contribute to elevated blood pressure [53]. Given the pivotal role of lipids in the development of the placenta, lipoprotein abnormalities could be related to the onset of pre-eclampsia [54]. A systematic review and meta-analysis including 74 studies revealed that women who develop pre-eclampsia show increased TC, non-HDL-C, and TG across all pregnancy trimesters, alongside lower HDL-C levels during the third trimester [13]. In particular, Gallos et al. found that elevated TG levels not only precede but are also associated with pre-eclampsia, highlighting the role of endothelial dysfunction, similar to their established impact in atherosclerosis [55].

During pregnancy, an increase in TG levels is a common physiological change [27]. TG do not directly cross the placenta and serve as an energy reservoir [36]. The most common secondary causes of hypertriglyceridemia during pregnancy other than inherited forms of dyslipidemias include conditions such as diabetes, medication use, obesity, and alcohol abuse [56]. High TG levels can also result in fetal complications, as reported by a prospective observational study involving 150 pregnant women with hypertriglyceridemia which found that neonates of these mothers often face complications such as prematurity, intrauterine growth restriction, and macrosomia [57]. A meta-analysis by Wang et al., which included 31,402 pregnancies, suggests that maternal high TG levels and low HDL-C levels during pregnancy are associated with increased birth weight, a higher risk of being large for the gestational age, and macrosomia, while showing a lower risk of being small for the gestational age. These associations were particularly pronounced in women who were overweight or obese before pregnancy [58].

### Lipid Metabolism and Pregestational Conditions

Pregnancy induces a myriad of physiological changes, including significant alterations in lipid metabolism, and preexisting conditions such as endocrinopathies, nephropathies and different forms of dyslipidemia can exacerbate lipid metabolic complications during pregnancy [59,60,61,62].

Pregestational diabetes mellitus is a significant risk factor for lipid metabolic disturbances during pregnancy [60]. In women with preexisting type 1 diabetes, the insulin deficiency, combined with the physiological insulin resistance of pregnancy, can exacerbate hyperglycemia and dyslipidemia [60,63]. This occurs because insulin plays a crucial role in regulating lipoprotein lipase activity, which is essential for the TG metabolism [28,64]. Such a process may be further exacerbated in type 2 diabetes mellitus [60]. Elevated levels of TG, LDL-C, and FFAs are commonly observed, increasing the risk of developing gestational hypertension, pre-eclampsia, and macrosomia [65,66]. Indeed, a recent systematic review reported that pregestational diabetes is associated with a higher incidence of pregnancy complications compared to GDM [67].

On the other hand, several preexisting endocrinopathies, including thyroid disorders and polycystic ovary syndrome (PCOS), can significantly impact lipid metabolism during pregnancy [68,69]. Hypothyroidism, for instance, is associated with an increase in serum cholesterol and TG levels due to decreased hepatic lipase activity and reduced clearance of lipoproteins [70,71]. This condition can result in complications like preterm delivery, as noted by Vella et al., who in a retrospective cohort study observed that hypothyroidism is associated with higher body weight and advanced maternal age [72]. PCOS, characterized by hyperandrogenism and insulin resistance, also poses challenges during pregnancy, as women with PCOS are more likely to experience elevated TGs and LDL-C, along with reduced HDL-C. These lipid abnormalities can contribute to an increased risk of cardiometabolic complications [73,74]. A large observational study found that women with PCOS had a higher risk of GDM, pre-eclampsia, and pre-term delivery compared to those without a PCOS diagnosis [75].

Chronic kidney disease (CKD), especially in its advanced stages, presents significant maternal and fetal risks, categorizing it as a high-risk condition during pregnancy [61]. This recognition has led to the emerging field of “obstetric nephrology”, highlighting the critical importance of managing kidney function during pregnancy [76]. The physiological changes in renal function during pregnancy, including increased clearance rates and proteinuria, elevate the risk of hypertensive disorders, notably pre-eclampsia, as well as fetal growth restriction and preterm delivery [61]. The severity of these adverse pregnancy outcomes escalates with the progression of CKD stages. Women with end-stage kidney disease who become pregnant typically have significantly reduced fertility; however, successful pregnancies are increasingly reported [77]. Managing pregnancy in dialysis patients requires an intensified dialysis regimen, often with daily sessions, to better control fluid balance, uremia, and hypertension, which are critical for minimizing maternal and fetal risks [78]. Studies suggest that more frequent and prolonged dialysis sessions are linked with better pregnancy outcomes, though the procedure still carries a high risk of complications, including preeclampsia, polyhydramnios, and fetal distress [78,79]. Moreover, CKD is associated with dyslipidemia, characterized by low HDL-C levels, elevated triglycerides, and normal LDL-C levels, but with a predominance of small, dense LDL particles, which can further exacerbate the risk of complications [80]. Patients undergoing peritoneal dialysis often exhibit a more atherogenic lipid profile compared to those on hemodialysis or non-dialysis-dependent patients, due to protein loss in the dialysate and the use of glucose-rich solutions, which promote atherosclerosis and increase cardiovascular risk [81]. 

Preexisting dyslipidemia can be exacerbated by pregnancy, especially considering the discontinuation of lipid-lowering therapy needed during this period [82]. Normally, pregnancy is associated with a physiological increase in lipid levels, with TG levels potentially doubling by the third trimester [27]. Table 1 shows the physiological changes in lipid parameters during pregnancy in comparison to the main primary dyslipidemias. In women with preexisting dyslipidemia, these changes can become pathological, leading to severe hypertriglyceridemia, which is characterized by TG > 1000 mg/dL and is often linked to genetic defects in lipid metabolism, such as familial combined hyperlipidemia, familial hypertriglyceridemia, hyperchylomicronemia, and familial dysbetalipoproteinemia [36]. Hypertriglyceridemia in pregnancy is associated with an increased risk of acute maternal complications, including acute pancreatitis and hyperviscosity syndrome [83]. Acute pancreatitis is an inflammatory condition of the pancreas that can cause severe abdominal pain and systemic illness [84]. Hyperviscosity syndrome, characterized by increased blood viscosity, can lead to impaired blood flow and tissue perfusion, raising the risk of complications such as thrombosis [85]. Additionally, high TG levels are associated with a higher risk of developing hyperlipidemia postpartum [86].

Elevated levels of LDL-C can contribute to the development of the ASCVD, thereby increasing the risk of cardiovascular events during pregnancy. Pregnant women naturally experience an increase in lipid levels, including those with preexisting dyslipidemia [62]. Special attention should be given to pregnant women with severe forms of familial heterozygous hypercholesterolemia (HeFH) or the rare genetic condition homozygous hypercholesterolemia (HoFH) [87], despite the few reported cases of the latter, as these conditions significantly impact the lipid profile [88]. According to a study by Amundsen et al., pregnant women with or without HeFH exhibited similar modifications in their lipid profiles [43] even if the absolute level was considerably greater in FH pregnancies. However, it is important to note that baseline LDL-C levels can be significantly higher in women with inherited dyslipidemias. Despite this, the risk of adverse outcomes (prematurity, low birth weight, and congenital malformations) in patients with HeFH is similar to that of women without this condition [39].

During pregnancy, it is important to also consider the lipoprotein(a) [Lp(a)], a known risk factor for arterial and venous thrombosis, due to its structural resemblance to plasminogen [89,90]. During pregnancy, a naturally reduced fibrinolytic activity occurs, and higher Lp(a) levels can further diminish fibrinolysis, which may lead to negative pregnancy outcomes [91]. Both pregnant women with previously high and normal Lp(a) levels may experience increases during pregnancy, and elevated Lp(a) levels can heighten the risk of maternal (i.e., pre-eclampsia) and neonatal (i.e., pre-term births) complications, although there are still conflicting results on this matter [89,91,92,93].

In summary, the interplay between pregnancy and preexisting conditions like endocrinopathies and chronic kidney disease significantly impacts lipid metabolism, increasing the risk of adverse outcomes for both the mother and the fetus. Understanding these dynamics is crucial for anticipating and managing potential complications during pregnancy, ensuring better maternal and fetal health outcomes. Improved awareness and targeted interventions can lead to more effective management strategies, ultimately enhancing the quality of care and reducing the risks associated with these conditions during pregnancy.

**Table 1:** Physiological changes in lipid parameters during pregnancy in comparison to main primary dyslipidemias.

	Pre-Gestation	During Pregnancy
**Healthy women [9,83]**	Normal lipid parameters	TC: +30–50% (≈250 mg/dL) LDL-C: +30–50% (140 mg/dL) HDL-C: +20–40% (≈65 mg/dL) TG: +50–100% (≈250 mg/dL)
**Primary dyslipidemias**		
Heterozygous familial hypercholesterolemia [94]	LDL-C: ≈200–250 mg/dL	LDL-C: +25–40% (≈250-350 mg/dL)
Homozygous familial hypercholesterolemia [95,96]	LDL-C: ≈500–600 mg/dL (untreated) ≈300–500 mg/dL (on therapy)	LDL-C: +20–60% (≈600–800 mg/dL, pre-apheresis)
Familial hyperchylomicronemia [83,97,98]	TG: range 1300–1500 mg/dL	TG: +350% (≈5000–7500 mg/dL)

## 4. Dietary and Lifestyle Approaches in Pregnancy

Nutrition is considered one of the most influential environmental factors that can affect embryonic and fetal development and also the health of mother and her fetus–neonate [99]. The maternal diet influences the intrauterine environment throughout an epigenetic mechanism that regulates gene expression in the unborn child, as an adaptation to various environmental conditions [100]. 

A healthy dietary pattern plays a crucial and determining role in ensuring a healthy process of growth and for mother, not only during pregnancy but also during the periconception period. Among different approaches, MD is considered a balanced and safety model for fetus and woman, preventing maternal and fetal diseases. In fact, according to the literature, MD should be suggested before, during, and after pregnancy [101,102]. As a useful approach in adults to counteract the development of metabolic syndrome, according to some authors, the MD during pregnancy could also protect the fetus from metabolic syndrome throughout the duration of life [100]. Based on different systematic reviews, higher maternal adherence to the MD during pregnancy seems to be protective against GDM, child adiposity, and may also lower the risk of postpartum depression [103,104]. MD appears also to be associated with lower hypertensive complications in pregnancy, higher fertility and chance of live birth after in vitro fertilization [105,106,107]. On the contrary, the Western diet, which significantly diverges from Mediterranean guidelines, is linked to a higher risk of developing pre-eclampsia in women during pregnancy [108]. Not only for the metabolic health of the fetus, but also for its neurological functioning, MD would seem to play a protective role. According to a recent prospective birth cohort study, greater maternal adherence to the MD would seem to be associated with better neurological development in the newborn and lower levels of Peptide C in the umbilical cord blood [16]. According to the authors, this association could be linked to the consumption of foods rich in beneficial nutrients typical of the Mediterranean pattern, such as omega-3 fatty acids, which are known for their neuroprotective virtues [109]. Although there are, however, studies confirming the correlation between MD and the mother’s carbohydrate metabolic functioning during pregnancy [110], there are currently insufficient data showing a link between the umbilical cord and the neurological development of the fetus [111].

The guidelines of the MD may vary slightly from country to country, as this dietary approach is adopted by various nations bordering the Mediterranean Sea and there is no a single MD pattern [112,113]. Among its defining elements, in addition to nutritional aspects, are strong references to cultural heritage, local traditions, and the agri-food sustainability of each region [114]. In general, the MD emphasizes a plant-based core and the consumption of so-called “good fats”, which can generally range from 35% to 45% of the total energy intake [115]. In particular, the lipid intake of the diet in Greece is around 40% of the total energy intake, with a share of monounsaturated fatty acids of about 17%, comparable to the amount consumed in Spain, due to the high consumption of olive oil, while in Portugal and also in Italy, fat consumption is shown to be lower [116]. While the relatively high fat content of MD might be suspected to increase weight gain and obesity, the results of the large randomized clinical trial PREDIMED indicate that such concerns are unfounded [117]. Indeed, subjects who had followed a diet enriched with extra virgin olive oil and nuts showed no significant increase in body weight or waist circumference compared to the control group [118]. Furthermore, they had a lower risk of developing type 2 diabetes and metabolic syndrome, all conditions closely linked to weight gain [119,120].

Given these premises, a dietary approach based on MD should be encouraged for the wellness of mother and child, as it is considered safe during pregnancy. 

The concept of a healthy diet not only concerns the quality of food but also the quantity and frequency of food intake. For this reason, the reference portions and consumption frequencies of the main food groups are outlined in the latest MD food pyramid [114]. In fact, a variety of food choices helps reduce the risk of developing nutritional deficiencies. These aspects become even more crucial when talking about diet in pregnant women. 

A deficient intake of macronutrients and micronutrients can negatively influence pregnancy outcomes and neonatal health, causing, for example, structural abnormalities of the fetus and long-term illnesses [99,121]. For these reasons, nutritional counseling during pregnancy should be considered as a cornerstone of prenatal care and health professionals must be informed about the increased nutritional needs of pregnant women, as the dietary requirements are significantly different from those of non-pregnant women. It is recommended to individualize nutritional counseling according to the woman’s cultural food choices, her access to food, her socio-economic status, and her pre-pregnancy body mass index [122]. However, in general, for a healthy and safe diet during pregnancy, it is recommended to have a varied and balanced diet that includes all food groups every day (Table 2) [123,124].

Although a total estimate of about 80,000 calories has been calculated to be necessary to cope with a full-term pregnancy, to cover the metabolic energy expenditure of the mother and the fetus and to ensure its growth in addition to that of the placenta, it is necessary to consider a woman’s customized calorie intake based on her pre-pregnancy BMI, age, lifestyle and activity level. There is indeed a great inter-individual variability in energy expenditure, linked not only to weight gain but also to a woman’s pre-pregnancy nutritional status [122]. According to pre-pregnancy BMI, the average weight gain of women has been established by international guidelines (Table 3) [124]. 

Energy expenditure starts to change not from the beginning of pregnancy, but from the second trimester to the third trimester, increasing by 350 kcal and 460 kcal daily in general, respectively, for a normal-weight pregnant woman. This additional intake will have to be scaled down if there is a reduction in physical activity and, according to national and international guidelines, should in any case not be applied universally to all body mass index (BMI) classes [123,124,125]. At the base of the MD pyramid are the non-nutritional elements that define the Mediterranean lifestyle, namely adequate rest, conviviality, and regular physical activity. In this regard, regular activity is defined as at least 30 min a day for 5 days [126]. 

The Academy of Nutrition and Dietetics also emphasizes the importance of leading an active lifestyle for all women of childbearing age who wish to become pregnant, as lifestyle choices greatly influence the health of the mother and the newborn. Good practices leading to a healthy gestation include an adequate body weight before pregnancy, controlled weight gain, and an adequate level of physical activity during pregnancy [127]. In women with a normal BMI before pregnancy, a prenatal nutrition and exercise protocol, regardless of intensity, has been shown to be beneficial in reducing pregnancy weight gain and normalizing postpartum body weight [128], as well as having a positive impact on blood glucose [129]. Furthermore, moderate physical activity during gestation is considered safe in low-risk pregnancies, as it does not lead to a risk of preterm delivery or miscarriage [130]. 

### 4.1. Macronutrients in Pregnancy

According to the Reference Intake Levels of Nutrients and Energy for the Italian Population (LARN), the requirement for protein intake increases during pregnancy, while carbohydrates and lipids remain nearly stable [123]. Protein intake should cover 20% of the daily energy requirement, and special attention should be paid to choosing proteins with a high biological value, such as those found in animal-derived food like eggs, milk and meat. The Protein Digestibility Corrected Amino Acid Score (PDCAAS) is used to evaluate the quality of food proteins. Animal products, which provide all nine essential amino acids, typically have a PDCAAS close to 1, which means that they provide a higher rate of indispensable amino acids in contrast to plant-based foods (PDCAAS below 0.7). However, by combining different plant-based foods with varying amino acid profiles, the overall quality of the protein can be improved [131]. 

Considering a total weight gain of about 12 kg in a pregnancy without complications, the population reference intake (PRI) of protein for the adult population is an increase of 1 g/day in the first trimester, 8 g/day in the second trimester, and 26 g/day in the third trimester. This increase supports protein synthesis in maternal tissues and fetal growth, which is highest in the last three months of gestation [123]. 

Similarly to the rest of the population, carbohydrates, mainly consumed as polysaccharides, represent the primary energy substrate for the body and should account for 45–60% of total energy intake. According to some authors, carbohydrate intake could reach 64% of total energy and should include up to nine servings of whole grains per day [122]. 

Lipid intake in pregnant women is comparable to that of non-pregnant women, in particular, the Italian reference intake levels indicate a range of lipids between 20 and 35% of total daily calories, of which saturated fats should not exceed 10% of total energy, with a limit on cholesterol intake as well, which should be less than 300 mg/day [123], but in other Mediterranean countries the reference percentages could vary and be wider [132]. Since it is important to maintain an adequate intake of essential fatty acids during pregnancy, the remaining lipid intake will consist of polyunsaturated fatty acids, omega-3 and omega-6 series, and monounsaturated fatty acids. Among the fatty acids of the omega-3 series, the most nutritionally interesting are docosahexaenoic acid (DHA) and eicosapentaenoic acid (EPA), which are considered beneficial in the development of the brain and retina nerve structures of the fetus, but also to improve cardiovascular health [122]. In particular, DHA is also known to have an anti-inflammatory effect by acting on Toll-like receptors and serving as a precursor to the anti-inflammatory lipid mediators, which inhibit the production of pro-inflammatory compounds from arachidonic acid [133]. DHA is primarily found in seafood. The body’s ability to convert other omega-3 fatty acids into DHA varies greatly between individuals due to differences in enzyme availability and metabolic function. Therefore, the most reliable way to meet DHA requirements is through the direct consumption of fish and seafood. Despite the known benefits of omega-3 fatty acids, it is common for the consumption of fish and other marine species to be avoided during pregnancy, due to the wide spread of toxic contaminants in the environment, including mercury [134]. In order to not run into harmful nutritional deficiencies, the pregnant woman should opt for small-sized fish, which are considered a good source of omega-3 fatty acids, such as sardines or anchovies, and at the same time reduce the consumption of fish with a high concentration of methylmercury, such as tuna, swordfish, shark, to a maximum of once a week [122,135]. However, according to LARN, there is no PRI of DHA in pregnancy, but only a reference range between 100 and 200 mg/day [123].

### 4.2. Micronutrients: Minerals and Vitamins

A healthy and balanced diet, which includes fresh foods such as fruit, vegetables and milk, guarantees the pregnant woman all the vitamins necessary to continue with her pregnancy. Vitamin D supplementation may not be necessary for all women, and its integration should be evaluated individually [135]. Also, vitamin A supplementation during pregnancy should be recommended only in case of deficiency [136]. 

The only exception concerns folic acid, which must be necessarily integrated at least up to 400 μg/day, for the prevention of neural tube defects, starting one month before conception and continuing during pregnancy up to the 12th week of gestation [137]. Pregnant women are recommended to take folic acid from fortified foods or daily supplements, in addition to consuming a diet rich in food sources of folate, such as citrus fruits, the dark green leaves of vegetables, nuts, and liver [122]. 

Similar to vitamins, a balanced dietary pattern provides all the minerals necessary for pregnancy. Calcium supplementation in women with normal calcium levels is not indicated [138].

Iron and iodine are the only other two micronutrients that typically require supplementation during pregnancy. In the case of iron deficiency, anemia supplementation is mandatory, and it is not sufficient to consume foods rich in iron, such as red meat, pork, fish and eggs [135]. 

The recommended intake level for iodine during pregnancy is 220–250 μg/day; however, in Italy, the quantity of iodine taken with food is not sufficient to guarantee the recommended daily intake, even when not pregnant. To overcome this nutritional deficiency, it is possible to resort to iodine-enriched salt and use it to replace common kitchen salt [139], since one gram of iodized salt contains 20–30 μg of iodine, or it is possible to ingest it through foods with a high iodine content or specific supplements for pregnancy [140]. Table 4 reports an overview of the specific nutritional needs in healthy pregnant women.

## 5. Nutritional Strategies for Managing Dyslipidemias in Pregnancy

According to the ESC/EAS guidelines [14] for cardiovascular prevention, the preferred dietary models are undoubtedly the Dietary Approaches to Stop Hypertension (DASH) and the MD. The latter is effective in preventing and treating several non-communicable diseases, such as cancer, diabetes, and, of course, cardiovascular problems [141]. In light of the metabolic changes that affect the liver and adipose tissue during pregnancy, determining notable variations in the circulating levels of triglycerides, FFA, cholesterol and phospholipids [142,143], the role of the MD can also be hypothesized in the context of the treatment of pregnancy complicated by dyslipidemia.

The effectiveness of the MD in treating hypertriglyceridemia lies in its components. This plant-based dietary model, typical of Mediterranean countries, is rich in bioactive compounds and notably low in simple and refined carbohydrate-rich foods, which are considered most harmful for triglyceride levels [144]. It provides adequate amounts of complex carbohydrates, throughout low-glycemic index foods, a modest amount of saturated fats with a higher proportion of polyunsaturated and monounsaturated fatty acids. Legumes, seasonal vegetables and fruits, unrefined cereals, nuts, fish, and lean meats are emphasized in the mediterranean pattern. Virgin olive oil, used primarily raw, serves as the main dietary fat [145,146]. 

Despite the fact that MD is widely recognized for its health benefits, there is still limited evidence specifically demonstrating its cholesterol-lowering effects during pregnancy. Nonetheless, a recent study investigating the correlation between adherence to the MD during pregnancy and maternal lipid markers found that higher adherence to MD was associated with a significant increased HDL-C levels, decreased LDL-C and TG levels and a more favorable LDL-C/HDL-C ratio at the 16th weeks of gestation, as well as a significant reduction in HDL-C and TG levels at the 34th weeks of gestation [147]. Additionally, a study conducted by Melero et al. indicated that women who followed the MD during pregnancy experienced improved lipid profiles postpartum [148]. 

In summary, the MD is optimal for managing dyslipidemias due to its well-known anti-atherogenic effect [19]; furthermore, MD represents the best dietary model in pregnancy from a nutritional point of view [149]. Moreover, according to evidence, regular but moderate exercise is more effective in improving lipid profile and it is considered safe during pregnancy [14,150].

Dietary management is also crucial for pregnant women with inherited forms of dyslipidemia. For instance, FH in pregnant patients necessitates the careful monitoring and adjustment of dietary intake to manage lipid levels effectively [42]. In addition to following general pregnancy dietary guidelines, these patients should adhere to either a cholesterol-restricted diet (<300 mg/day) or a cholesterol-free diet (<50 mg/day), as both have been shown to decrease LDL-C levels [151,152]. Emphasizing the consumption of foods high in fiber, low in saturated fats, and low in cholesterol is still essential for optimal lipid control [14,153].

For subjects with hypertriglyceridemia and hyperchylomicronemic syndrome, in addition to the dietary recommendations previously outlined, it is essential to limit sugar intake, as sugars can worsen TG levels [144]. Furthermore, alcohol consumption should be completely avoided, not only because it can significantly increase TG levels but also because it aligns with general pregnancy guidelines to prevent fetal alcohol spectrum disorder [154,155]. Patients with severe hypertriglyceridemia, particularly those with inherited forms, may benefit from a low-fat diet consisting of less than 15–20% of total energy intake [156]. Omega-3 fatty acids should be the primary fat source, as they are not included into chylomicrons, reduce lipogenesis, and enhance fatty acid oxidation [157]. However, strict adherence to a very low-fat diet may lead to weight loss, which can have adverse effects on both maternal and fetal health [158]. In some cases, the consumption of medium-chain triglycerides is recommended, as they are directly absorbed into the bloodstream and utilized for energy, potentially providing a safer lipid alternative without contributing to elevated TG levels [159,160].

Overall, dietary management is an indispensable component of the multidisciplinary approach to treating pregnant women with dyslipidemia, especially the inherited forms. The complexity of these latter conditions requires not only adhering to general pregnancy dietary guidelines but also implementing specific dietary strategies. This approach helps to manage lipid levels effectively and prevents complications associated with elevated TG and cholesterol. Moreover, working closely with a dietitian to customize these nutritional strategies ensures the health and safety of both mother and fetus throughout pregnancy. Through such tailored interventions, the risks associated with dyslipidemia can be significantly mitigated, highlighting the critical role of personalized dietary counseling in prenatal care.

## 6. Pharmacological Approach

In a normal pregnancy, the woman shows a 30–50% (≈250 mg/dL) physiological increase in plasma TC and a two-three fold increase in plasma TG (≈250 mg/dL), but in pathological conditions, such as FH or hypertriglyceridemia (both familial and secondary forms), severe hyperlipoproteinemia is a possibility, with negative consequences for mother and pregnancy outcomes [42,83,151,161,162]. Here, we present first the available treatments for severe hypercholesterolemia and then for severe hypertriglyceridemia in pregnancy.

### 6.1. Familial Hypercholesterolemia: Treatment in Pregnancy

Women affected by FH, in particular HeFH not controlled and HoFH, are generally treated by long-term cholesterol-lowering therapy. A combination of both pharmacologic intervention and lipoprotein apheresis (LA) may be fundamental for HoFH women or FH women at high cardiovascular risk. The advent of pregnancy raises the problem of controlling the obvious increase in TC and LDL-C, considering the recommendation of stopping the usual hypolipemic drugs. For these reasons, there is a general consensus of avoiding unplanned pregnancy to allow the pre-pregnancy evaluation of the individual cardiovascular risk profile and to choose the best treatment in any single patient. Unfortunately, the available literature fails to show definitive results of clinical studies, including dyslipidemic pregnant women treated with hypolipemic drugs; the current results derive from experimental animal studies or observations of case reports on the unintentional use of drugs in pregnancy.

#### 6.1.1. Bile Acid Sequestrants

Bile Acid Sequestrants (BASs) are the only drugs approved for treating dyslipidemia in pregnancy: the mechanism of action is localized in the intestinal lumen, so it does not enter the maternal circulation and cannot reach the fetus [163]. They reduce the TC by 15% and LDL-C by approximately 20–30% [151]. BAS are not currently indicated as the starting isolated medications for the FH treatment and are associated with statin and ezetimibe [164]. Thus, their efficacy is limited and it is associated with side effects that may exacerbate symptoms already present in pregnancy. 

#### 6.1.2. Fibrates, Nicotinic Acid, Ezetimibe

Fibrates are not recommended in pregnancy because they have been associated with fetal malformations in animal studies and included in old category C of the US Food and Drug Administration (FDA) and the Pregnancy and Lactation Labeling Rule (PLLR) [163]. They should only be considered when the benefits clearly outweigh the risks [14]. The same considerations are indicated for Nicotinic acid (niacin) use in pregnancy; thus, it is not known whether this drug can damage the fetus (FDA old category C and PLLR). Ezetimibe is a potent and selective inhibitor of cholesterol intestinal absorption, which decreases biliary cholesterol secretion and promotes the synthesis of LDL receptors, with a subsequent reduction in serum LDL-C [165,166]. Ezetimibe is more currently used in association with statins in FH patients. Few animal studies in pregnancy showed dangerous effects on skeletal system [167]; no well-conducted clinical studies have been reported (FDA old category C).

#### 6.1.3. Statins

Statins, HMG-CoA reductase inhibitors, are the most common used drugs in the treatment of dyslipidemia and are the first-line therapy in FH patients exhibiting substantial benefits in primary and secondary prevention of cardiovascular events [164,168]. As a class, statins are contraindicated (FDA old category X) during pregnancy and lactation [164,168,169]. This recommendation derives from animal studies which demonstrated fetal harm and reduced birth weight [163,170]. Concerning the human studies, potential adverse birth effects have been shown in case reports and small cohort studies after lipophilic statin exposure [163,171,172]; however, pregnant mothers treated with hydrophilic statins (pravastatin) did not present any fetal malformation [172]. In recent years, several studies [173,174,175,176] found that the prevalence of congenital abnormalities in mothers exposed to statins was similar to the unexposed population [163,177,178]. However, other observations reported a higher risk of miscarriage [179], low birth weight and preterm labor [179]. 

The FDA, on the basis of the new evidence about the safety of statins in pregnancy, recommended changing the limits of the statin prescription. FDA requested removal of strongest warning against using cholesterol-lowering statins during all pregnancies [180]. Notwithstanding, the discontinuation of statins during pregnancy and lactation should in general continue to be recommended [181].

So, the question of if it is still appropriate to contraindicate statins in all pregnancies is widely debated. Some experts suggest continuing statin in pregnant high-CV-risk patients, including women with HoFH and FH with previous ASCVD, after the first trimester [82,88,168,182]. Another consideration in favor of the use of statins in pregnancy derives from few human clinical trials aimed to determine whether a hydrophilic statin may be used to prevent pre-eclampsia [170,174,183].

In summary, healthcare professionals and patients might make individual decisions discussing benefits and risks in the single case.

#### 6.1.4. New Therapies

New hypocholesterolemic drugs have been recently utilized alone or in association with statins [82]. 

PCSK9 (proprotein convertase subtilisin: kexin type 9) inhibitors, according to their biologic mechanism of action in cellular proliferation and differentiation, must be considered with caution in pregnancy. Studies on primates show contrasting results [184,185], and evidence in human pregnancies even not completely negative is still insufficient [14,82,164,168,184,186,187]. A very recent study with an innovative genetic technique provides evidence to avoid the use of these medications in pregnancy due to the potential risk of fetal malformations [188]. 

Bempedoic acid, a new LDL non-statin-lowering drug has been introduced for use in 2020, and is utilized alone or in association with low dose statins; since very limited data are present on its use during pregnancy, it must be discontinued in pregnancy and lactation [82]

Evinacumab, an angiopoietin-like protein (ANGPTL)3 inhibitor, is a monoclonal antibody, and crosses the placental barrier. The available human data are insufficient to evaluate the risk for adverse fetal or maternal outcomes during pregnancy [82].

Inclisiran is a novel small interfering RNA-based therapy (anti-sense). It must be administered only twice a year and presents a safe profile in controlled study; however, we do not have available data during pregnancy, and it must be discontinued [82].

Lomitapide reduces VLDL release and VLDL-mediated triglyceride secretion. It may cause fetal toxicity and data during human pregnancy are not available. It must be discontinued in pregnancy [82,182]. 

Mipomersen lowers LDL-C by reducing hepatic VLDL production and therefore can act independently of the presence of the LDL receptor. Animal studies do not demonstrate teratogenicity [189]. There are no controlled data for pregnant women. Mipomersen should be used in pregnancy only if clearly needed (FDA pregnancy old category: B) [182,190].

### 6.2. Lipoprotein Apheresis for Managing Dyslipidemia in Pregnancy

LA is an extracorporeal removal of cholesterol-carrying lipoproteins utilizing different techniques (adsorption, differential filtration, precipitation) [191]. LA is an efficient and safe procedure recommended in HoFH, and in severe HeFH pregnant women at high ASCVD risk [88,164,168,186,192,193].

Evidence-based guidelines for the LA use during pregnancy are still lacking; thus, clinical criteria are based on available case reports, case series and scientific societies opinions. The American Society of Apheresis (ASFA) encourages the LA use in HoFH and HeFH because it offers recognized benefits (category I-grade 1A and category II-grade 1A, respectively) [193]. Moreover, LA is the only approved treatment for elevated Lp(a) [194].

LA provides the possibility of reducing LDL-C levels to an optimal goal [195] and has been demonstrated as safe in pregnancy for the mother and the fetus [88,196,197]. LA, in reducing LDL-C levels [197], reduces both the maternal and fetal ASCVD risk. Moreover, therapeutic apheresis has pleiotropic effects involving pro-inflammatory and anti-inflammatory factors, and hemodynamic changes such as vasodilation and increased blood flow [198]. These effects are favorable for maternal–fetus placental circulation, preventing pre-eclampsia and intrauterine growth restriction. LA should be continued or initiated during pregnancy in women with HoFH, especially in those with established ASCVD and in whom LDL-cholesterol levels are not at the guideline-recommended goal; similar advice applies to women with severe HeFH [164]. There are no specific indications in pregnancy regarding the different selective LA methods and the frequency of treatments [88,191,199]. All known LA methods seem to have equal efficacy, and the recommended frequency of LA in pregnancy is one to two times per week according to the target LDL-C level obtained [88,197].

Postpartum is a crucial period in FH mothers: since pharmacological treatments are contraindicated (lipid-lowering drugs pass into the milk) and lactation is strongly encouraged for the baby and mother, LDL apheresis is still indicated to allow breastfeeding for as long as needed. The use of LA in pregnancy requires specialized clinical infrastructure and a multidisciplinary team of different specialties, so it cannot be available in all medical services; even if FDA and experts opinions have considered statins safe in severe high ASCVD risk FH pregnancies, LA still represents the more efficient and safe option for mother and fetus. Due to the paucity of the evidence linked to the small number of case reports, we cannot foresee the general guidelines in the near future. Therefore, at the moment, individualized multidisciplinary strategy has to be considered as the gold standard for the treatment of these high-risk patients. 

### 6.3. Severe Hypertriglyceridemia: Treatment in Pregnancy

Gestational hypertriglyceridemia defined as TG levels above the 95th percentile is found in non-genetic conditions or in patients with genetic mutations in enzymes involved in the metabolic process of TG (familial chylomicronaemia syndrome or familial hypertriglyceridemia) [200]. In these patients, hypertriglyceridemia-induced pancreatitis is associated with a maternal mortality rate as high as 20% [200,201]. Pharmacotherapy becomes necessary if TG levels increase above 500 mg/dL [200].

Lowering plasma TG levels prevents the onset of acute pancreatitis, as this risk increases progressively as TG levels rise [202,203,204]. Treatment options for TG reduction during pregnancy are limited. Although there are codified guidelines, such as those of the Endocrine Society [205], on the management of severe hypertriglyceridemia in the nonpregnant state; in pregnancy, the current recommendations are only based on observational data.

#### 6.3.1. Statins

Statin treatment is recommended as the first drug of choice for reducing ASCVD risk in high-risk individuals with hypertriglyceridaemia [206]. Statins are mainly used to reduce LDL-C, but they also reduce TG levels. For this reason, statins may reduce the risk of pancreatitis [203]. 

Statins have always been considered contraindicated in pregnancy because of animal data showing their teratogenic potential [183,207,208,209]. However, recent large observational cohort studies have found no teratogenic effect of statins [208,210]. Statins are not the drugs of first choice in the treatment of hypertriglyceridemia unless there is a high cardiovascular risk. Physicians may consider their use during pregnancy in the above patients and preferably after the first trimester. Because statins are now known to interfere with some of the pathologic mechanisms underlying pre-eclampsia, a reevaluation of their potential use in the prevention of this obstetric complication would be desirable [211]. Breastfeeding is not recommended in patients taking statins [212,213]. 

#### 6.3.2. Fibrates

Fibrate therapy reduces serum TG levels by 50–70% [214,215], although favorable clinical effects are limited. Fibrates are associated with muscle toxicity [216,217] and this risk is greater in patients also taking statins [218,219,220]. Fibrates cross the human blood–placental barrier [221]. According to the FDA, they are designated in old category C (unclear teratogenicity) because they have shown teratogenic effects in animal models [151]. However, there are two case reports on the use of gemfibrozil and fenofibrate after the first trimester without teratogenic or toxic effects [157,222]. In pregnant patients with severe hypertriglyceridemia at risk for pancreatitis, the use of fenofibrate or gemfibrozil starting in the second trimester may be considered [36,223]. Fibrates should be considered only if TG levels should be >500 mg/dL despite the use of omega-3 fatty acids, dietary restrictions, and if plasmapheresis cannot be performed [200]. 

There is no relevant published information on the use of fenofibrate or gemfibrozil during breastfeeding. Breastfeeding in women taking fibrates is contraindicated, because of the potential risk of altered lipid metabolism in the child [224,225]. So, in patients with severe hypertriglyceridemia, it is recommended to consider whether it is more appropriate to discontinue breastfeeding or discontinue the drug.

#### 6.3.3. Omega-3

Omega-3 fatty acids reduce TG levels by about 20–50%, with an effect dependent on baseline TG values and directly proportional to the dose used [226,227,228,229]. High-dose omega-3 fatty acids can be used safely during pregnancy [230]. Indeed, the increased intake of them during pregnancy is associated with improved maternal–fetal outcomes [231]: reduced rates of intrauterine growth restriction [232,233], preterm birth [234,235,236], perinatal death, and improved neurocognitive outcomes in the offspring [232]. At the same time, it could increase the risk of large children for gestational age [233]; and atrial fibrillation for the mother [237,238,239]. 

In women with severe hypertriglyceridemia and at risk for pancreatitis, the use of omega-3 fatty acid at high doses during pregnancy may be effective and safe [158] and may prevent the development of acute pancreatitis [240]. The daily dose of omega-3 fatty acids to reduce TG levels is 4 g [228]. Omega-3 fatty acid therapy can also be safely taken during breastfeeding.

#### 6.3.4. Nicotinic Acid (Niacin)

Niacin is a water-soluble vitamin involved in the synthesis and metabolism of carbohydrates, fatty acids, and proteins. Niacin inhibits diacylglycerol transferase-2, leading to a reduction in TG [157]. At high doses of 1500–2000 mg per day it reduces TG levels by 15–25% [215]. Compared with fibrates, niacin reduces TG levels to a lesser extent and has more side effects [241]. Niacin, like all water-soluble vitamins, crosses the placenta [242]. It is not known whether niacin at therapeutic doses for lipid reduction is harmful to the developing fetus, as there are no studies evaluating its safety in pregnancy [157,243]. For these reasons, niacin should be discontinued in pregnancy. The suspension of breastfeeding is strongly recommended in patients taking niacin for the treatment of hypertriglyceridemia.

#### 6.3.5. Bile Acid Sequestrants

BASs are the only lipid-lowering drugs currently accepted in pregnancy [83,151,244]. These drugs do not pass into the systemic circulation and are safe for the fetus. However, they can lead to increased triglyceride levels and VLDL, worsening the preexisting condition. For this reason, this class of drugs are contraindicated in patients with severe hypertriglyceridemia (>1000 mg/dL) [205,245].

#### 6.3.6. New Therapies

Emerging therapies reduce the activity of apolipoprotein C-III (APOC3) and angiopoietin-like protein ANGPTL3, resulting in increased TG clearance. There are currently no studies on the use of these new therapies in the treatment of patients with hypertriglyceridemia in pregnancy.

#### 6.3.7. Plasmapheresis

Therapeutic apheresis can be used to reduce TG levels or to prevent hypertriglyceridemia-induced complications during pregnancy [200,246,247,248]. Aphaeretic treatment results in a rapid decrease in TG levels in a short period of time [249,250,251]. The percentage reduction in serum levels of TG after a single procedure is about 49–80% [83,252,253,254,255,256,257,258]. Many studies and clinical cases have evaluated the safety and efficacy of plasma exchange in pregnant patients [163,259,260,261,262,263,264]. Plasmapheresis is effective and safe for the treatment of pregnant women with severe hypertriglyceridemia and pancreatitis [158]. It should be considered in asymptomatic pregnant women with fasting triglyceride levels > 1000 mg/dL or in pregnant women with clinical signs and symptoms of pancreatitis and triglyceride levels > 500 mg/dL despite lifestyle changes and pharmacologic therapy. In addition, it should be discontinued when triglyceride levels are <400 mg/dL or when signs or symptoms of pancreatitis disappear [200]. 

The use of plasmapheresis is limited due to the rather high costs and the limited availability of the procedure [251]. For these reasons, unfortunately, it is a treatment option not available in all centers.

## 7. Conclusions

To summarize, the management of dyslipidemia during pregnancy presents unique challenges, necessitating a careful balance between maternal and fetal health. The physiological changes in lipid metabolism during pregnancy, particularly the increase in TG levels and LDL-C levels, can exacerbate pre-existing conditions such as FH and hypertriglyceridemia. Effective management strategies include lifestyle modifications, dietary interventions, and, when necessary, pharmacological treatments. Moreover, clinical trials investigating the efficacy of dietary patterns in pregnant women with dyslipidemia are still limited. However, in inherited forms of dyslipidemia (i.e., familial hyperchylomicronemia), personalized dietary adjustments are pivotal for successful pregnancy outcomes. While emerging therapies and lipoprotein apheresis offer promising options for severe cases, their availability, high costs, and the limited research hinder their widespread use. 

Despite these advancements, the current body of research is insufficient to establish comprehensive guidelines that address the full spectrum of dyslipidemia in pregnancy. The distinctive physiological changes during pregnancy complicate the management of lipid levels, and the interplay between maternal and fetal health adds further complexity. Thus, more clinical trials are needed to explore the efficacy and safety of dietary patterns, pharmacological treatments, and other therapeutic interventions in this population. Additionally, it is important to explore the feasibility and cost-effectiveness of these interventions in different populations, taking into account ethnicity, healthcare access and socioeconomic factors. Close monitoring and a personalized approach involving a multidisciplinary team are essential to optimize outcomes for both mother and child. Further research and clinical trials are needed to establish definitive guidelines and expand the safe and effective treatment options for dyslipidemic pregnant women. 

## Figures and Tables

**Table 2:** Minimum recommended daily servings for each food group.

Food Group	Serving in g [123]	Serving/Day
Bread, cereals, rice, pasta, etc.	50 to 80 g	9
Vegetables	80 to 200 g	4
Fruits	150 g	3
Milk, yogurt, fresh cheese	100 to 125 g	2–3
Meat, fish, dried beans, eggs, nuts:	50 to 150 g	1–2

**Table 3:** Advisable weight gain through pregnancy.

BMI Pre-Pregnancy	Weight Gain in the Second and Third Trimester on Average in Single Pregnancy (Expressed in kg/Week)	Desirable Weight Gain at the End of Single Gestation (Expressed in kg)	Desirable Weight Gain at the End of Twin Gestation (Expressed in kg)
**Underweight** **(BMI: <18.5 kg/m^2^)**	0.51 (0.44–0.58)	12.5–18	Not available
**Normal weight** **(BMI: 18.5–24.9 kg/m^2^)**	0.42 (0.35–0.50)	11.5–16	17–24.5
**Overweight** **(BMI: 25–29.9 kg/m^2^)**	0.28 (0.23–0.33)	7–11.5	14–22.7
**Obesity** **(BMI: > 30 kg/m^2^)**	0.22 (1.17–0.27)	5–9	11.5–19

Adapted from Institute of Medicine (US) and National Research Council (US) Committee to Reexamine IOM Pregnancy Weight Guidelines. *Weight Gain During Pregnancy: Reexamining the Guidelines*. Rasmussen KM, Yaktine AL, editors. Washington (DC): National Academies Press (US); 2009.

**Table 4:** Overview of specific nutritional needs in healthy pregnant women.

**Calories**	+350 kcal/Day in the II Trimester +460 kcal/Day in the III Trimester
**Protein**	+1 g/day in the I trimester +8 g/day in the II trimester +26 g in the III trimester
**Carbohydrates**	45–60% of total kcal, with an intake of simple sugars not exceeding 10–15%
**Lipids**	≈35% of total kcal, saturated fatty acids <10%. DHA +100–200 mg/day
**Fiber**	25–30 g/day
**Water**	+350 mL/day (compared to the pre-pregnancy period)
**Sodium**	1.5 g/day, the adequate intake corresponds to that defined for the general adult population
**Calcium**	1200 mg/day
**Iron**	27 mg/day
**Iodine**	200–250 μg/day
**Folic acid**	400 µg/day or 500 µg/day in the case of women who have given birth to fetuses with neural tube defects or who have a history of neurological malformations

## Data Availability

All data are available in the text of the manuscript.

## References

[1] Roeters Van Lennep J.E., Tokgözoǧlu L.S., Badimon L., Dumanski S.M., Gulati M., Hess C.N., Holven K.B., Kavousi M., Kaylkçloǧlu M., Lutgens E. (2023). Women, Lipids, and Atherosclerotic Cardiovascular Disease: A Call to Action from the European Atherosclerosis Society. Eur. Heart J..

[2] Liu T., Zhao D., Qi Y. (2022). Global Trends in the Epidemiology and Management of Dyslipidemia. J. Clin. Med..

[3] Yanai H., Yoshida H. (2021). Secondary Dyslipidemia: Its Treatments and Association with Atherosclerosis. Glob. Health Med..

[4] Cho S.M.J., Lee H.J., Shim J.S., Song B.M., Kim H.C. (2020). Associations between Age and Dyslipidemia Are Differed by Education Level: The Cardiovascular and Metabolic Diseases Etiology Research Center (CMERC) Cohort. Lipids Health Dis..

[5] Formisano E., Pasta A., Cremonini A.L., Di Lorenzo I., Sukkar S.G., Pisciotta L. (2021). Effects of a Mediterranean Diet, Dairy, and Meat Products on Different Phenotypes of Dyslipidemia: A Preliminary Retrospective Analysis. Nutrients.

[6] Schienkiewitz A., Truthmann J., Ernert A., Wiegand S., Schwab K.O., Scheidt-Nave C. (2019). Age, Maturation and Serum Lipid Parameters: Findings from the German Health Survey for Children and Adolescents. BMC Public Health.

[7] Sharma J., McAlister J., Aggarwal N.R., Wei J., Mehta P.K., Quesada O., Mattina D., Scott N.S., Michos E.D., Mahmoud Z. (2022). Evaluation and Management of Blood Lipids through a Woman’s Life Cycle. Am. J. Prev. Cardiol..

[8] Upmeier E., Lavonius S., Heinonen P., Viitanen M., Isoaho H., Arve S., Lehtonen A. (2011). Longitudinal Changes in Serum Lipids in Older People the Turku Elderly Study 1991–2006. Age Ageing.

[9] Mulder J.W.C.M., Kusters D.M., van Lennep J.E.R., Hutten B.A. (2024). Lipid Metabolism during Pregnancy: Consequences for Mother and Child. Curr. Opin. Lipidol..

[10] Li C., Li X., Wu D., Chen Q., Xiao Z., Wen D., Zhai L., Jia L. (2021). Influence of Dietary Behaviors on Dyslipidemia in Pregnant Women and Its Effects on Physical Development of Fetuses and Infants: A Bidirectional Cohort Study. Nutrients.

[11] Graves M., Howse K., Pudwell J., Smith G.N. (2019). Pregnancy-Related Cardiovascular Risk Indicators: Primary Care Approach to Postpartum Management and Prevention of Future Disease. Can. Fam. Physician.

[12] Tabacu C., Manolea M.-M., Novac L., Dijmarescu A.L., Boldeanu M.V. (2021). Maternal Lipid Profile as a Risk Factor for Gestational Diabetes Mellitus in Obese Women. Curr. Health Sci. J..

[13] Spracklen C.N., Smith C.J., Saftlas A.F., Robinson J.G., Ryckman K.K. (2014). Maternal Hyperlipidemia and the Risk of Preeclampsia: A Meta-Analysis. Am. J. Epidemiol..

[14] Mach F., Baigent C., Catapano A.L., Koskina K.C., Casula M., Badimon L., Chapman M.J., De Backer G.G., Delgado V., Ference B.A. (2019). 2019 ESC/EAS Guidelines for the Management of Dyslipidaemias: Lipid Modification to Reduce Cardiovascular Risk. Atherosclerosis.

[15] Makarem N., Chau K., Miller E.C., Gyamfi-Bannerman C., Tous I., Booker W., Catov J.M., Haas D.M., Grobman W.A., Levine L.D. (2022). Association of a Mediterranean Diet Pattern With Adverse Pregnancy Outcomes Among US Women. JAMA Netw. Open.

[16] Dai F.C., Wang P., Li Q., Zhang L., Yu L.J., Wu L., Tao R.X., Zhu P. (2023). Mediterranean Diet during Pregnancy and Infant Neurodevelopment: A Prospective Birth Cohort Study. Front. Nutr..

[17] Richardson L.A., Izuora K., Basu A. (2022). Mediterranean Diet and Its Association with Cardiovascular Disease Risk Factors: A Scoping Review. Int. J. Environ. Res. Public Health.

[18] Escobar C., Anguita M., Arrarte V., Barrios V., Cequier Á., Cosín-Sales J., Egocheaga I., López de Sa E., Masana L., Pallarés V. (2020). Recommendations to Improve Lipid Control. Consensus Document of the Spanish Society of Cardiology. Rev. Esp. Cardiol. (Engl. Ed.).

[19] Pasta A., Formisano E., Cremonini A.L., Maganza E., Parodi E., Piras S., Pisciotta L. (2020). Diet and Nutraceutical Supplementation in Dyslipidemic Patients: First Results of an Italian Single Center Real-World Retrospective Analysis. Nutrients.

[20] Mauricio R., Khera A. (2022). Statin Use in Pregnancy: Is It Time For a Paradigm Shift?. Circulation.

[21] Bohn M.K., Adeli K. (2022). Physiological and Metabolic Adaptations in Pregnancy: Importance of Trimester-Specific Reference Intervals to Investigate Maternal Health and Complications. Crit. Rev. Clin. Lab. Sci..

[22] Zeng Z., Liu F., Li S. (2017). Metabolic Adaptations in Pregnancy: A Review. Ann. Nutr. Metab..

[23] Herrera E. (2002). Lipid Metabolism in Pregnancy and Its Consequences in the Fetus and Newborn. Endocrine.

[24] Lain K.Y., Catalano P.M. (2007). Metabolic Changes in Pregnancy. Clin. Obstet. Gynecol..

[25] Rung E., Friberg P.A., Shao R., Larsson D.G.J., Nielsen E.C., Svensson P.A., Carlsson B., Carlsson L.M.S., Billig H. (2005). Progesterone-Receptor Antagonists and Statins Decrease de Novo Cholesterol Synthesis and Increase Apoptosis in Rat and Human Periovulatory Granulosa Cells in Vitro. Biol. Reprod..

[26] Duttaroy A.K., Basak S. (2022). Maternal Fatty Acid Metabolism in Pregnancy and Its Consequences in the Feto-Placental Development. Front. Physiol..

[27] Ghio A., Bertolotto A., Resi V., Volpe L., Di Cianni G. (2011). Triglyceride Metabolism in Pregnancy. Adv. Clin. Chem..

[28] Goldberg I.J., Eckel R.H., Abumrad N.A. (2009). Regulation of Fatty Acid Uptake into Tissues: Lipoprotein Lipase- and CD36-Mediated Pathways. J. Lipid Res..

[29] Bowman C.E., Arany Z., Wolfgang M.J. (2021). Regulation of Maternal-Fetal Metabolic Communication. Cell Mol. Life Sci..

[30] Herrera E., Desoye G. (2016). Maternal and Fetal Lipid Metabolism under Normal and Gestational Diabetic Conditions. Horm. Mol. Biol. Clin. Investig..

[31] Melchior J.T., Swertfeger D.K., Morris J., Street S.E., Warshak C.R., Welge J.A., Remaley A.T., Catov J.M., Davidson W.S., Woollett L.A. (2021). Pregnancy Is Accompanied by Larger High Density Lipoprotein Particles and Compositionally Distinct Subspecies. J. Lipid Res..

[32] Piechota W., Staszewski A. (1992). Reference Ranges of Lipids and Apolipoproteins in Pregnancy. Eur. J. Obstet. Gynecol. Reprod. Biol..

[33] Wang C., Kong L., Yang Y., Wei Y., Zhu W., Su R., Lin L., Yang H. (2018). Recommended Reference Values for Serum Lipids during Early and Middle Pregnancy: A Retrospective Study from China. Lipids Health Dis..

[34] Perrine C.G., Nelson J.M., Corbelli J., Scanlon K.S. (2016). Lactation and Maternal Cardio-Metabolic Health. Annu. Rev. Nutr..

[35] Lu F., Ferriero D.M., Jiang X. (2022). Cholesterol in Brain Development and Perinatal Brain Injury: More than a Building Block. Curr. Neuropharmacol..

[36] Wong B., Ooi T.C., Keely E. (2015). Severe Gestational Hypertriglyceridemia: A Practical Approach for Clinicians. Obstet. Med..

[37] Dalfrà M.G., Burlina S., Ragazzi E., Pastrolin S., Sartore G., Lapolla A. (2024). Lipid Profile in Women of Different Ethnicity with Gestational Diabetes: Relationship with Fetal Growth. J. Diabetes Investig..

[38] Wang Y., Chen Z., Zhang F. (2022). Association between Maternal Lipid Levels during Pregnancy and Delivery of Small for Gestational Age: A Systematic Review and Meta-Analysis. Front. Pediatr..

[39] Toleikyte I., Retterstøl K., Leren T.P., Iversen P.O. (2011). Pregnancy Outcomes in Familial Hypercholesterolemia: A Registry-Based Study. Circulation.

[40] Pasta A., Cremonini A.L., Formisano E., Fresa R., Bertolini S., Pisciotta L. (2020). Long Term Follow-up of Genetically Confirmed Patients with Familial Hypercholesterolemia Treated with First and Second-Generation Statins and Then with PCSK9 Monoclonal Antibodies. Atherosclerosis.

[41] Casula M., Gazzotti M., Capra M.E., Olmastroni E., Galimberti F., Catapano A.L., Pederiva C., Anesi A., Arca M., Auricchio R. (2023). Refinement of the Diagnostic Approach for the Identification of Children and Adolescents Affected by Familial Hypercholesterolemia: Evidence from the LIPIGEN Study. Atherosclerosis.

[42] Graham D.F., Raal F.J. (2021). Management of Familial Hypercholesterolemia in Pregnancy. Curr. Opin. Lipidol..

[43] Amundsen Å.L., Khoury J., Iversen P.O., Bergei C., Ose L., Tonstad S., Retterstøl K. (2006). Marked Changes in Plasma Lipids and Lipoproteins during Pregnancy in Women with Familial Hypercholesterolemia. Atherosclerosis.

[44] Abu-Awwad S.A., Craina M., Boscu L., Bernad E., Ciordas P.D., Marian C., Iurciuc M., Abu-Awwad A., Iurciuc S., Bernad B. (2023). Lipid Profile Variations in Pregnancies with and without Cardiovascular Risk: Consequences for Both Mother and Newborn. Children.

[45] Li G., Kong L., Zhang L., Fan L., Su Y., Rose J.C., Zhang W. (2015). Early Pregnancy Maternal Lipid Profiles and the Risk of Gestational Diabetes Mellitus Stratified for Body Mass Index. Reprod. Sci..

[46] Ryckman K.K., Spracklen C.N., Smith C.J., Robinson J.G., Saftlas A.F. (2015). Maternal Lipid Levels during Pregnancy and Gestational Diabetes: A Systematic Review and Meta-Analysis. BJOG.

[47] Rahnemaei F.A., Pakzad R., Amirian A., Pakzad I., Abdi F. (2021). Effect of Gestational Diabetes Mellitus on Lipid Profile: A Systematic Review and Meta-Analysis. Open Med (Wars)..

[48] O’Malley E.G., Reynolds C.M.E., Killalea A., O’Kelly R., Sheehan S.R., Turner M.J. (2020). Maternal Obesity and Dyslipidemia Associated with Gestational Diabetes Mellitus (GDM). Eur. J. Obstet. Gynecol. Reprod. Biol..

[49] Hardy O.T., Czech M.P., Corvera S. (2012). What Causes the Insulin Resistance Underlying Obesity?. Curr. Opin. Endocrinol. Diabetes Obes..

[50] Stadler J.T., Lackner S., Mörkl S., Trakaki A., Scharnagl H., Borenich A., Wonisch W., Mangge H., Zelzer S., Meier-Allard N. (2021). Obesity Affects HDL Metabolism, Composition and Subclass Distribution. Biomedicines.

[51] Formisano E., Proietti E., Borgarelli C., Sukkar S.G., Albertelli M., Boschetti M., Pisciotta L. (2024). The Impact of Overweight on Lipid Phenotype in Different Forms of Dyslipidemia: A Retrospective Cohort Study. J. Endocrinol. Investig..

[52] Wojcik-Baszko D., Charkiewicz K., Laudanski P. (2018). Role of Dyslipidemia in Preeclampsia—A Review of Lipidomic Analysis of Blood, Placenta, Syncytiotrophoblast Microvesicles and Umbilical Cord Artery from Women with Preeclampsia. Prostaglandins Other Lipid Mediat..

[53] Roberts J.M., Escudero C. (2012). The Placenta in Preeclampsia. Pregnancy Hypertens..

[54] Yang Y., Wang Y., Lv Y., Ding H. (2022). Dissecting the Roles of Lipids in Preeclampsia. Metabolites.

[55] Gallos I.D., Sivakumar K., Kilby M.D., Coomarasamy A., Thangaratinam S., Vatish M. (2013). Pre-Eclampsia Is Associated with, and Preceded by, Hypertriglyceridaemia: A Meta-Analysis. BJOG.

[56] Kleess L.E., Janicic N. (2019). Severe hypertriglyceridemia in pregnancy: A case report and review of the literature. AACE Clin. Case Rep..

[57] Dash S., Tiwari M., Dash P., Kar K., Mohakud N.K. (2022). Complications of Hypertriglyceridemia in Pregnancy and Its Impact on Neonates: A Hospital-Based Study from Odisha. Cureus.

[58] Wang J., Moore D., Subramanian A., Cheng K.K., Toulis K.A., Qiu X., Saravanan P., Price M.J., Nirantharakumar K. (2018). Gestational Dyslipidaemia and Adverse Birthweight Outcomes: A Systematic Review and Meta-Analysis. Obes. Rev..

[59] Calina D., Docea A.O., Golokhvast K.S., Sifakis S., Tsatsakis A., Makrigiannakis A. (2019). Management of Endocrinopathies in Pregnancy: A Review of Current Evidence. Int. J. Environ. Res. Public Health.

[60] Alexopoulos A.S., Blair R., Peters A.L. (2019). Management of Preexisting Diabetes in Pregnancy: A Review. JAMA.

[61] Hui D., Hladunewich M.A. (2019). Chronic Kidney Disease and Pregnancy. Obstet. Gynecol..

[62] Baumfeld Y., Novack L., Wiznitzer A., Sheiner E., Henkin Y., Sherf M., Novack V. (2015). Pre-Conception Dyslipidemia Is Associated with Development of Preeclampsia and Gestational Diabetes Mellitus. PLoS ONE.

[63] Huhtala M., Rönnemaa T., Tertti K. (2023). Insulin Resistance Is Associated with an Unfavorable Serum Lipoprotein Lipid Profile in Women with Newly Diagnosed Gestational Diabetes. Biomolecules.

[64] Wu S.A., Kersten S., Qi L. (2021). Lipoprotein Lipase and Its Regulators: An Unfolding Story. Trends Endocrinol. Metab..

[65] Toescu V., Nuttall S.L., Martin U., Nightingale P., Kendall M.J., Brydon P., Dunne F. (2004). Changes in Plasma Lipids and Markers of Oxidative Stress in Normal Pregnancy and Pregnancies Complicated by Diabetes. Clin. Sc. (Lond.).

[66] Oros Ruiz M., Perejón López D., Serna Arnaiz C., Siscart Viladegut J., Àngel Baldó J., Sol J. (2024). Maternal and Foetal Complications of Pregestational and Gestational Diabetes: A Descriptive, Retrospective Cohort Study. Sci. Rep..

[67] Malaza N., Masete M., Adam S., Dias S., Nyawo T., Pheiffer C. (2022). A Systematic Review to Compare Adverse Pregnancy Outcomes in Women with Pregestational Diabetes and Gestational Diabetes. Int. J. Environ. Res. Public Health.

[68] Jiang X., Lu X., Cai M., Liu Y., Guo Y. (2022). Impact of Dyslipidemia on the Cumulative Pregnancy Outcomes after First Ovarian Stimulation. Front. Endocrinol..

[69] Morón-Díaz M., Saavedra P., Alberiche-Ruano M.P., Rodríguez-Pérez C.A., López-Plasencia Y., Marrero-Arencibia D., González-Lleó A.M., Boronat M. (2021). Correlation between TSH Levels and Quality of Life among Subjects with Well-Controlled Primary Hypothyroidism. Endocrine.

[70] Liu H., Peng D. (2022). Update on Dyslipidemia in Hypothyroidism: The Mechanism of Dyslipidemia in Hypothyroidism. Endocr. Connect..

[71] Moon J.H., Kim H.J., Kim H.M., Choi S.H., Lim S., Park Y.J., Jang H.C., Cha B.S. (2013). Decreased Expression of Hepatic Low-Density Lipoprotein Receptor–Related Protein 1 in Hypothyroidism: A Novel Mechanism of Atherogenic Dyslipidemia in Hypothyroidism. Thyroid.

[72] Vella K., Vella S., Savona-Ventura C., Vassallo J. (2022). Thyroid Dysfunction in Pregnancy—A Retrospective Observational Analysis of a Maltese Cohort. BMC Pregnancy Childbirth.

[73] Palomba S., Falbo A., Chiossi G., Muscogiuri G., Fornaciari E., Orio F., Tolino A., Colao A., La Sala G.B., Zullo F. (2014). Lipid Profile in Nonobese Pregnant Women with Polycystic Ovary Syndrome: A Prospective Controlled Clinical Study. Steroids.

[74] Wild R.A., Rizzo M., Clifton S., Carmina E. (2011). Lipid Levels in Polycystic Ovary Syndrome: Systematic Review and Meta-Analysis. Fertil. Steril..

[75] Farland L.V., Stern J.E., Liu C.L., Cabral H.J., Coddington C.C., Diop H., Dukhovny D., Hwang S., Missmer S.A. (2022). Polycystic Ovary Syndrome and Risk of Adverse Pregnancy Outcomes: A Registry Linkage Study from Massachusetts. Hum. Reprod..

[76] August P. (2012). Obstetric Nephrology: Pregnancy and the Kidney--Inextricably Linked. Clin. J. Am. Soc. Nephrol..

[77] Oliverio A.L., Hladunewich M.A. (2020). End Stage Kidney Disease and Dialysis in Pregnancy. Adv. Chronic Kidney Dis..

[78] Piccoli G.B., Zakharova E., Attini R., Hernandez M.I., Guillien A.O., Alrukhaimi M., Liu Z.H., Ashuntantang G., Covella B., Cabiddu G. (2018). Pregnancy in Chronic Kidney Disease: Need for Higher Awareness. A Pragmatic Review Focused on What Could Be Improved in the Different CKD Stages and Phases. J. Clin. Med..

[79] Hladunewich M.A., Hou S., Odutayo A., Cornelis T., Pierratos A., Goldstein M., Tennankore K., Keunen J., Hui D., Chan C.T. (2014). Intensive Hemodialysis Associates with Improved Pregnancy Outcomes: A Canadian and United States Cohort Comparison. J. Am. Soc. Nephrol..

[80] Visconti L., Benvenga S., Lacquaniti A., Cernaro V., Bruzzese A., Conti G., Buemi M., Santoro D. (2016). Lipid Disorders in Patients with Renal Failure: Role in Cardiovascular Events and Progression of Chronic Kidney Disease. J. Clin. Transl. Endocrinol..

[81] Lluesa J.H., López-Romero L.C., Monzó J.J.B., Marugán M.R., Boyano I.V., Rodríguez-Espinosa D., Gómez-Bori A., Orient A.S., Such R.D., Perez P.S. (2022). Lipidic Profiles of Patients Starting Peritoneal Dialysis Suggest an Increased Cardiovascular Risk beyond Classical Dyslipidemia Biomarkers. Sci. Rep..

[82] Lloyd-Jones D.M., Morris P.B., Ballantyne C.M., Birtcher K.K., Covington A.M., DePalma S.M., Minissian M.B., Orringer C.E., Smith S.C., Waring A.A. (2022). 2022 ACC Expert Consensus Decision Pathway on the Role of Nonstatin Therapies for LDL-Cholesterol Lowering in the Management of Atherosclerotic Cardiovascular Disease Risk: A Report of the American College of Cardiology Solution Set Oversight Committee. J. Am. Coll. Cardiol..

[83] Bashir M., Navti O.B., Frcog M., Ahmed B., Konje J.C., Frcog F. (2023). Hyperlipidaemia and Severe Hypertriglyceridaemia in Pregnancy. Obstet. Gynaecol..

[84] Szatmary P., Grammatikopoulos T., Cai W., Huang W., Mukherjee R., Halloran C., Beyer G., Sutton R. (2022). Acute Pancreatitis: Diagnosis and Treatment. Drugs.

[85] Gangopadhyay D., Roy M., Laha S., Nandi D., Sengupta R., Chattopadhyay A. (2022). Hyperviscosity Syndrome Revisited. Ann. Pediatr. Cardiol..

[86] Zhu Y., Zhu H., Dang Q., Yang Q., Huang D., Zhang Y., Cai X., Yu H. (2021). Changes in Serum TG Levels during Pregnancy and Their Association with Postpartum Hypertriglyceridemia: A Population-Based Prospective Cohort Study. Lipids Health Dis..

[87] D’Erasmo L., Bini S., Casula M., Gazzotti M., Bertolini S., Calandra S., Tarugi P., Averna M., Iannuzzo G., Fortunato G. (2024). Contemporary Lipid-Lowering Management and Risk of Cardiovascular Events in Homozygous Familial Hypercholesterolaemia: Insights from the Italian LIPIGEN Registry. Eur. J. Prev. Cardiol..

[88] Blaha M., Lanska M., Blaha V., Boudys L., Zak P. (2015). Pregnancy in Homozygous Familial Hypercholesterolemia--Importance of LDL-Apheresis. Atheroscler. Suppl..

[89] Fanshawe A.E., Ibrahim M. (2013). The Current Status of Lipoprotein (a) in Pregnancy: A Literature Review. J. Cardiol..

[90] Cremonini A.L., Pasta A., Carbone F., Visconti L., Casula M., Elia E., Bonaventura A., Liberale L., Bertolotto M., Artom N. (2022). Lipoprotein(a) Modulates Carotid Atherosclerosis in Metabolic Syndrome. Front. Mol. Biosci..

[91] Sattar N., Clark P., Greer I.A., Shepherd J., Packard C.J. (2000). Lipoprotein (a) Levels in Normal Pregnancy and in Pregnancy Complicated with Pre-Eclampsia. Atherosclerosis.

[92] Golawski M., Lejawa M., Osadnik T., Mickiewicz A., Gierlotka M., Jozwiak J., Pawlas N., Banach M. (2023). Genetically Determined Lipoprotein(a) Levels Do Not Cause an Increased Risk of Preeclampsia—A Two-Sample Mendelian Randomization Study. Eur. Heart J..

[93] Romagnuolo I., Sticchi E., Attanasio M., Grifoni E., Cioni G., Cellai A.P., Abbate R., Fatini C. (2016). Searching for a Common Mechanism for Placenta-Mediated Pregnancy Complications and Cardiovascular Disease: Role of Lipoprotein(a). Fertil. Steril..

[94] Rutherford J.D. (2011). Maternal Heterozygous Familial Hypercholesterolemia and Its Consequences for Mother and Child. Circulation.

[95] Cuchel M., Bruckert E., Ginsberg H.N., Raal F.J., Santos R.D., Hegele R.A., Kuivenhoven J.A., Nordestgaard B.G., Descamps O.S., Steinhagen-Thiessen E. (2014). Homozygous Familial Hypercholesterolaemia: New Insights and Guidance for Clinicians to Improve Detection and Clinical Management. A Position Paper from the Consensus Panel on Familial Hypercholesterolaemia of the European Atherosclerosis Society. Eur. Heart J..

[96] Fahed A.C., Nassar A.H. (2012). Pregnancy in a Woman with Homozygous Familial Hypercholesterolemia Not on Low-Density Lipoprotein Apheresis. AJP Rep..

[97] Coronado Arroyo J.C., Concepción Zavaleta M.J., García Villasante E.J., Kcomt Lam M., Concepción Urteaga L.A., Zavaleta Gutiérrez F.E. (2021). Familial Chylomicronemia Syndrome-Induced Acute Necrotizing Pancreatitis during Pregnancy. RBGO Gynecol. Obstet..

[98] Jaafar B., Chaaya J.A., Ammar S., Salti I. (2023). Acute Pancreatitis in Pregnancy and Familial Chylomicronemia Syndrome: Case Report and Literature Review. Metab. Target. Organ. Damage.

[99] Cetin I., Berti C., Calabrese S. (2010). Role of Micronutrients in the Periconceptional Period. Hum. Reprod. Update.

[100] Lorite Mingot D., Gesteiro E., Bastida S., Sánchez-Muniz F.J. (2017). Epigenetic Effects of the Pregnancy Mediterranean Diet Adherence on the Offspring Metabolic Syndrome Markers. J. Physiol. Biochem..

[101] Yee L.M., Silver R.M., Haas D.M., Parry S., Mercer B.M., Iams J., Wing D., Parker C.B., Reddy U.M., Wapner R.J. (2020). Quality of Periconceptional Dietary Intake and Maternal and Neonatal Outcomes. Am. J. Obstet. Gynecol..

[102] De Giuseppe R., Bocchi M., Maffoni S., Del Bo E., Manzoni F., Cerbo R.M., Porri D., Cena H. (2021). Mediterranean Diet and Lifestyle Habits during Pregnancy: Is There an Association with Small for Gestational Age Infants? An Italian Single Centre Experience. Nutrients.

[103] Eckl M.R., Brouwer-Brolsma E.M., Küpers L.K. (2021). Maternal Adherence to the Mediterranean Diet during Pregnancy: A Review of Commonly Used a Priori Indexes. Nutrients.

[104] Flor-Alemany M., Migueles J.H., Alemany-Arrebola I., Aparicio V.A., Baena-García L. (2022). Exercise, Mediterranean Diet Adherence or Both during Pregnancy to Prevent Postpartum Depression—GESTAFIT Trial Secondary Analyses. Int. J. Environ. Res. Public Health.

[105] Schoenaker D.A.J.M., Soedamah-Muthu S.S., Callaway L.K., Mishra G.D. (2015). Prepregnancy Dietary Patterns and Risk of Developing Hypertensive Disorders of Pregnancy: Results from the Australian Longitudinal Study on Women’s Health. Am. J. Clin. Nutr..

[106] Winter H.G., Rolnik D.L., Mol B.W.J., Torkel S., Alesi S., Mousa A., Habibi N., Silva T.R., Oi Cheung T., Thien Tay C. (2023). Can Dietary Patterns Impact Fertility Outcomes? A Systematic Review and Meta-Analysis. Nutrients.

[107] Ricci E., Bravi F., Noli S., Somigliana E., Cipriani S., Castiglioni M., Chiaffarino F., Vignali M., Gallotti B., Parazzini F. (2019). Mediterranean Diet and Outcomes of Assisted Reproduction: An Italian Cohort Study. Am. J. Obstet. Gynecol..

[108] Esquivel M.K. (2023). Nutritional Status and Nutrients Related to Pre-Eclampsia. Am. J. Lifestyle Med..

[109] Gómez-Pinilla F. (2008). Brain Foods: The Effects of Nutrients on Brain Function. Nat. Rev. Neurosci..

[110] Guasch-Ferré M., Willett W.C. (2021). The Mediterranean Diet and Health: A Comprehensive Overview. J. Intern. Med..

[111] Wang P., Xie J., Jiao X.C., Ma S.S., Liu Y., Yin W.J., Tao R.X., Hu H.L., Zhang Y., Chen X.X. (2021). Maternal Glycemia During Pregnancy and Early Offspring Development: A Prospective Birth Cohort Study. J. Clin. Endocrinol. Metab..

[112] Dominguez L.J., Veronese N., Di Bella G., Cusumano C., Parisi A., Tagliaferri F., Ciriminna S., Barbagallo M. (2023). Mediterranean Diet in the Management and Prevention of Obesity. Exp. Gerontol..

[113] Romaguera D., Bamia C., Pons A., Tur J.A., Trichopoulou A. (2009). Food Patterns and Mediterranean Diet in Western and Eastern Mediterranean Islands. Public Health Nutr..

[114] Bach-Faig A., Berry E.M., Lairon D., Reguant J., Trichopoulou A., Dernini S., Medina F.X., Battino M., Belahsen R., Miranda G. (2011). Mediterranean Diet Pyramid Today. Science and Cultural Updates. Public Health Nutr..

[115] Martínez-González M.Á., Hershey M.S., Zazpe I., Trichopoulou A. (2017). Transferability of the Mediterranean Diet to Non-Mediterranean Countries. What Is and What Is Not the Mediterranean Diet. Nutrients.

[116] Cruz J.A.A. (2000). Dietary Habits and Nutritional Status in Adolescents over Europe--Southern Europe. Eur. J. Clin. Nutr..

[117] Estruch R., Ros E., Salas-Salvadó J., Covas M.-I., Corella D., Arós F., Gómez-Gracia E., Ruiz-Gutiérrez V., Fiol M., Lapetra J. (2018). Primary Prevention of Cardiovascular Disease with a Mediterranean Diet Supplemented with Extra-Virgin Olive Oil or Nuts. N. Engl. J. Med..

[118] Estruch R., Martínez-González M.A., Corella D., Salas-Salvadó J., Fitó M., Chiva-Blanch G., Fiol M., Gómez-Gracia E., Arós F., Lapetra J. (2019). Effect of a High-Fat Mediterranean Diet on Bodyweight and Waist Circumference: A Prespecified Secondary Outcomes Analysis of the PREDIMED Randomised Controlled Trial. Lancet Diabetes Endocrinol..

[119] Babio N., Toledo E., Estruch R., Ros E., Martínez-González M.A., Castañer O., Bulló M., Corella D., Arós F., Gómez-Gracia E. (2014). Mediterranean Diets and Metabolic Syndrome Status in the PREDIMED Randomized Trial. CMAJ.

[120] Salas-Salvadó J., Bulló M., Estruch R., Ros E., Covas M.-I., Ibarrola-Jurado N., Corella D., Arós F., Gómez-Gracia E., Ruiz-Gutiérrez V. (2014). Prevention of Diabetes with Mediterranean Diets: A Subgroup Analysis of a Randomized Trial. Ann. Intern. Med..

[121] Mousa A., Naqash A., Lim S. (2019). Macronutrient and Micronutrient Intake during Pregnancy: An Overview of Recent Evidence. Nutrients.

[122] Kominiarek M.A., Rajan P. (2016). Nutrition Recommendations in Pregnancy and Lactation. Med. Clin. N. Am..

[123] (2014). *LARN Livelli Di Assunzione Di Riferimento Di Nutrienti Ed Energia per La Popolazione Italiana*; SICS Editore; IV Revisione. https://air.uniud.it/handle/11390/1041183?mode=complete.

[124] Rasmussen K.M., Yaktine A.L. (2009). Weight Gain During Pregnancy: Reexamining the Guidelines.

[125] EFSA Panel on Dietetic Products, Nutrition and Allergies (NDA) (2013). Scientific Opinion on Dietary Reference Values for Energy. EFSA J..

[126] Serra-Majem L., Tomaino L., Dernini S., Berry E.M., Lairon D., de la Cruz J.N., Bach-Faig A., Donini L.M., Medina F.X., Belahsen R. (2020). Updating the Mediterranean Diet Pyramid towards Sustainability: Focus on Environmental Concerns. Int. J. Environ. Res. Public Health.

[127] Koletzko B., Cremer M., Flothkötter M., Graf C., Hauner H., Hellmers C., Kersting M., Krawinkel M., Przyrembel H., Röbl-Mathieu M. (2018). Diet and Lifestyle Before and During Pregnancy—Practical Recommendations of the Germany-Wide Healthy Start—Young Family Network. Geburtshilfe Frauenheilkd..

[128] Ruchat S.M., Davenport M.H., Giroux I., Hillier M., Batada A., Sopper M.M., Hammond J.M.S., Mottola M.F. (2012). Nutrition and Exercise Reduce Excessive Weight Gain in Normal-Weight Pregnant Women. Med. Sci. Sports Exerc..

[129] Deierlein A.L., Siega-Riz A.M., Evenson K.R. (2012). Physical Activity during Pregnancy and Risk of Hyperglycemia. J. Womens Health.

[130] Barakat R., Zhang D., Sánchez-Polán M., Silva-José C., Gil-Ares J., Franco E. (2023). Is Exercise during Pregnancy a Risk for Gestational Age and Preterm Delivery? Systematic Review and Meta-Analysis. J. Clin. Med..

[131] EFSA (European Food Safety Authority) (2017). Dietary Reference Values for Nutrients Summary Report. EFSA Support. Publ..

[132] Eilander A., Harika R.K., Zock P.L. (2015). Intake and Sources of Dietary Fatty Acids in Europe: Are Current Population Intakes of Fats Aligned with Dietary Recommendations?. Eur. J. Lipid Sci. Technol..

[133] Danielewicz H., Myszczyszyn G., Dębińska A., Myszkal A., Boznański A., Hirnle L. (2017). Diet in Pregnancy—More than Food. Eur. J. Pediatr..

[134] Genuis S.J., Genuis R.A. (2016). Preconception Care: A New Standard of Care within Maternal Health Services. Biomed. Res. Int..

[135] Alibrandi A., Zirilli A., Le Donne M., Giannetto C., Lanfranchi M., De Pascale A., Politi C., Incognito G.G., Ercoli A., Granese R. (2024). Association between Fish Consumption during Pregnancy and Maternal and Neonatal Outcomes: A Statistical Study in Southern Italy. J. Clin. Med..

[136] McGuire S. (2012). WHO Guideline: Vitamin A Supplementation in Pregnant Women. Geneva: WHO, 2011; WHO Guideline: Vitamin A Supplementation in Postpartum Women. Geneva: WHO, 2011. Adv. Nutr..

[137] Morse N.L. (2012). Benefits of Docosahexaenoic Acid, Folic Acid, Vitamin D and Iodine on Foetal and Infant Brain Development and Function Following Maternal Supplementation during Pregnancy and Lactation. Nutrients.

[138] (2013). World Health Organization Guideline: Calcium Supplementation in Pregnant Women.

[139] Iacone R., Iaccarino Idelson P., Russo O., Donfrancesco C., Krogh V., Sieri S., Macchia P.E., Formisano P., Lo Noce C., Palmieri L. (2021). Iodine Intake from Food and Iodized Salt as Related to Dietary Salt Consumption in the Italian Adult General Population. Nutrients.

[140] Alexander E.K., Pearce E.N., Brent G.A., Brown R.S., Chen H., Dosiou C., Grobman W.A., Laurberg P., Lazarus J.H., Mandel S.J. (2017). 2017 Guidelines of the American Thyroid Association for the Diagnosis and Management of Thyroid Disease During Pregnancy and the Postpartum. Thyroid.

[141] Sofi F., Macchi C., Abbate R., Gensini G.F., Casini A. (2014). Mediterranean Diet and Health Status: An Updated Meta-Analysis and a Proposal for a Literature-Based Adherence Score. Public Health Nutr..

[142] Herrera E., Ortega-Senovilla H. (2010). Disturbances in Lipid Metabolism in Diabetic Pregnancy—Are These the Cause of the Problem?. Best Pract. Res. Clin. Endocrinol. Metab..

[143] Barbour L.A., Hernandez T.L. (2018). Maternal Lipids and Fetal Overgrowth: Making Fat from Fat. Clin. Ther..

[144] Luna-Castillo K.P., Olivares-Ochoa X.C., Hernández-Ruiz R.G., Llamas-Covarrubias I.M., Rodríguez-Reyes S.C., Betancourt-Núñez A., Vizmanos B., Martínez-López E., Muñoz-Valle J.F., Márquez-Sandoval F. (2022). The Effect of Dietary Interventions on Hypertriglyceridemia: From Public Health to Molecular Nutrition Evidence. Nutrients.

[145] Mentella M.C., Scaldaferri F., Ricci C., Gasbarrini A., Miggiano G.A.D. (2019). Cancer and Mediterranean Diet: A Review. Nutrients.

[146] Zelber-Sagi S., Salomone F., Mlynarsky L. (2017). The Mediterranean Dietary Pattern as the Diet of Choice for Non-Alcoholic Fatty Liver Disease: Evidence and Plausible Mechanisms. Liver Int..

[147] Flor-Alemany M., Acosta-Manzano P., Migueles J.H., Baena-García L., Aranda P., Aparicio V.A. (2023). Association of Mediterranean Diet Adherence during Pregnancy with Maternal and Neonatal Lipid, Glycemic and Inflammatory Markers: The GESTAFIT Project. Matern. Child. Nutr..

[148] Melero V., Arnoriaga M., Barabash A., Valerio J., del Valle L., Martin O’Connor R., de Miguel M.P., Diaz J.A., Familiar C., Moraga I. (2023). An Early Mediterranean-Based Nutritional Intervention during Pregnancy Reduces Metabolic Syndrome and Glucose Dysregulation Rates at 3 Years Postpartum. Nutrients.

[149] Amati F., Hassounah S., Swaka A. (2019). The Impact of Mediterranean Dietary Patterns During Pregnancy on Maternal and Offspring Health. Nutrients.

[150] Ribeiro M.M., Andrade A., Nunes I. (2021). Physical Exercise in Pregnancy: Benefits, Risks and Prescription. J. Perinat. Med..

[151] Eapen D.J., Valiani K., Reddy S., Sperling L. (2012). Management of Familial Hypercholesterolemia during Pregnancy: Case Series and Discussion. J. Clin. Lipidol..

[152] Glueck C.J., Christopher C., Tsang R.C., Mellies M.J. (1980). Cholesterol-Free Diet and the Physiologic Hyperlipidemia of Pregnancy in Familial Hypercholesterolemia. Metabolism.

[153] Trautwein E.A., McKay S. (2020). The Role of Specific Components of a Plant-Based Diet in Management of Dyslipidemia and the Impact on Cardiovascular Risk. Nutrients.

[154] Popova S., Dozet D., Shield K., Rehm J., Burd L. (2021). Alcohol’s Impact on the Fetus. Nutrients.

[155] Do Rego A.T., Klop B., Birnie E., Elte J.W.F., Cachofeiro Ramos V., Walther Alvarez-Sala L.A., Castro Cabezas M. (2013). Diurnal Triglyceridemia in Relation to Alcohol Intake in Men. Nutrients.

[156] Williams L., Rhodes K.S., Karmally W., Welstead L.A., Alexander L., Sutton L. (2018). Familial Chylomicronemia Syndrome: Bringing to Life Dietary Recommendations throughout the Life Span. J. Clin. Lipidol..

[157] Goldberg A.S., Hegele R.A. (2012). Severe Hypertriglyceridemia in Pregnancy. J. Clin. Endocrinol. Metab..

[158] Nguyen N.T., Nath P.V., Mai V.Q., Shakir M.K.M., Hoang T.D. (2021). Treatment of Severe Hypertriglyceridemia During Pregnancy With High Doses of Omega-3 Fatty Acid and Plasmapheresis. AACE Clin. Case Rep..

[159] Papamandjaris A.A., Macdougall D.E., Jones P.J.H. (1998). Medium Chain Fatty Acid Metabolism and Energy Expenditure: Obesity Treatment Implications. Life Sci..

[160] Jadhav H.B., Annapure U.S. (2023). Triglycerides of Medium-Chain Fatty Acids: A Concise Review. J. Food Sci. Technol..

[161] Nangrahary M., Graham D.F., Pang J., Barnett W., Watts G.F. (2023). Familial Hypercholesterolaemia in Pregnancy: Australian Case Series and Review. Aust. N. Z. J. Obstet. Gynaecol..

[162] Soma-Pillay P., Nelson-Piercy C., Tolppanen H., Mebazaa A. (2016). Physiological Changes in Pregnancy. Cardiovasc. J. Afr..

[163] Mauri M., Calmarza P., Ibarretxe D. (2021). Dyslipemias and Pregnancy, an Update. Clin. Investig. Arterioscler..

[164] Watts G.F., Gidding S.S., Hegele R.A., Raal F.J., Sturm A.C., Jones L.K., Sarkies M.N., Al-Rasadi K., Blom D.J., Daccord M. (2023). International Atherosclerosis Society Guidance for Implementing Best Practice in the Care of Familial Hypercholesterolaemia. Nat. Rev. Cardiol..

[165] Bruckert E., Giral P., Tellier P. (2003). Perspectives in Cholesterol-Lowering Therapy: The Role of Ezetimibe, a New Selective Inhibitor of Intestinal Cholesterol Absorption. Circulation.

[166] Wang Y., Zhan S., Du H., Li J., Khan S.U., Aertgeerts B., Guyatt G., Hao Q., Bekkering G., Li L. (2022). Safety of Ezetimibe in Lipid-Lowering Treatment: Systematic Review and Meta-Analysis of Randomised Controlled Trials and Cohort Studies. BMJ Med..

[167] Parker B.A., Thompson P.D. (2012). Effect of statins on skeletal muscle: Exercise, myopathy, and muscle outcomes. Exerc. Sport Sci. Rev..

[168] Cuchel M., Raal F.J., Hegele R.A., Al-Rasadi K., Arca M., Averna M., Bruckert E., Freiberger T., Gaudet D., Harada-Shiba M. (2023). 2023 Update on European Atherosclerosis Society Consensus Statement on Homozygous Familial Hypercholesterolaemia: New Treatments and Clinical Guidance. Eur. Heart J..

[169] Harada-Shiba M., Ohtake A., Sugiyama D., Tada H., Dobashi K., Matsuki K., Minamino T., Yamashita S., Yamamoto Y. (2023). Guidelines for the Diagnosis and Treatment of Pediatric Familial Hypercholesterolemia 2022. J. Atheroscler. Thromb..

[170] Döbert M., Varouxaki A.N., Mu A.C., Syngelaki A., Ciobanu A., Akolekar R., De Paco Matallana C., Cicero S., Greco E., Singh M. (2021). Pravastatin Versus Placebo in Pregnancies at High Risk of Term Preeclampsia. Circulation.

[171] Edison R.J., Muenke M. (2004). Central Nervous System and Limb Anomalies in Case Reports of First-Trimester Statin Exposure. N. Engl. J. Med..

[172] Ghidini A., Sicherer S., Willner J. (1992). Congenital Abnormalities (VATER) in Baby Born to Mother Using Lovastatin. Lancet.

[173] Bateman B.T., Hernandez-Diaz S., Fischer M.A., Seely E.W., Ecker J.L., Franklin J.M., Desai R.J., Allen-Coleman C., Mogun H., Avorn J. (2015). Statins and Congenital Malformations: Cohort Study. BMJ.

[174] Maierean S.M., Mikhailidis D.P., Toth P.P., Grzesiak M., Mazidi M., Maciejewski M., Banach M. (2018). The Potential Role of Statins in Preeclampsia and Dyslipidemia during Gestation: A Narrative Review. Expert. Opin. Investig. Drugs.

[175] Vahedian-Azimi A., Makvandi S., Banach M., Reiner Ž., Sahebkar A. (2021). Fetal Toxicity Associated with Statins: A Systematic Review and Meta-Analysis. Atherosclerosis.

[176] Vahedian-Azimi A., Bianconi V., Makvandi S., Banach M., Mohammadi S.M., Pirro M., Sahebkar A. (2021). A Systematic Review and Meta-Analysis on the Effects of Statins on Pregnancy Outcomes. Atherosclerosis.

[177] Wu T., Shi Y., Zhu B., Li D., Li Z., Zhao Z., Zhang Y. (2024). Pregnancy-Related Adverse Events Associated with Statins: A Real-World Pharmacovigilance Study of the FDA Adverse Event Reporting System (FAERS). Expert. Opin. Drug Saf..

[178] Karalis D.G., Hill A.N., Clifton S., Wild R.A. (2016). The Risks of Statin Use in Pregnancy: A Systematic Review. J. Clin. Lipidol..

[179] Chang J.C., Chen Y.J., Chen I.C., Lin W.S., Chen Y.M., Lin C.H. (2021). Perinatal Outcomes After Statin Exposure During Pregnancy. JAMA Netw. Open.

[180] FDA Requests Removal of Strongest Warning against Using Cholesterol-Lowering Statins during Pregnancy; Still Advises Most Pregnant Patients Should Stop Taking Statins. https://www.fda.gov/drugs/drug-safety-and-availability/fda-requests-removal-strongest-warning-against-using-cholesterol-lowering-statins-during-pregnancy.

[181] Pollack P.S., Shields K.E., Burnett D.M., Osborne M.J., Cunningham M.L., Stepanavage M.E. (2005). Pregnancy Outcomes after Maternal Exposure to Simvastatin and Lovastatin. Birth Defects Res. A Clin. Mol. Teratol..

[182] Botha T.C., Pilcher G.J., Wolmarans K., Blom D.J., Raal F.J. (2018). Statins and Other Lipid-Lowering Therapy and Pregnancy Outcomes in Homozygous Familial Hypercholesterolaemia: A Retrospective Review of 39 Pregnancies. Atherosclerosis.

[183] Zarek J., Koren G. (2014). The Fetal Safety of Statins: A Systematic Review and Meta-Analysis. J. Obstet. Gynaecol. Can..

[184] Grześk G., Dorota B., Wołowiec Ł., Wołowiec A., Osiak J., Kozakiewicz M., Banach J. (2022). Safety of PCSK9 Inhibitors. Biomed. Pharmacother..

[185] Pasta A., Cremonini A.L., Pisciotta L., Buscaglia A., Porto I., Barra F., Ferrero S., Brunelli C., Rosa G.M. (2020). PCSK9 Inhibitors for Treating Hypercholesterolemia. Expert. Opin. Pharmacother..

[186] Harada-Shiba M., Arai H., Ohmura H., Okazaki H., Sugiyama D., Tada H., Dobashi K., Matsuki K., Minamino T., Yamashita S. (2023). Guidelines for the Diagnosis and Treatment of Adult Familial Hypercholesterolemia 2022. J. Atheroscler. Thromb..

[187] Macchi C., Iodice S., Persico N., Ferrari L., Cantone L., Greco M.F., Ischia B., Dozio E., Corsini A., Sirtori C.R. (2021). Maternal Exposure to Air Pollutants, PCSK9 Levels, Fetal Growth and Gestational Age—An Italian Cohort. Environ. Int..

[188] Ardissino M., Slob E.A.W., Reddy R.K., Morley A.P., Schuermans A., Hill P., Williamson C., Honigberg M.C., De Marvao A., Ng F.S. (2024). Genetically Proxied Low-Density Lipoprotein Cholesterol Lowering via PCSK9-Inhibitor Drug Targets and Risk of Congenital Malformations. Eur. J. Prev. Cardiol..

[189] Gouni-Berthold I., Berthold H.K. (2015). Mipomersen and Lomitapide: Two New Drugs for the Treatment of Homozygous Familial Hypercholesterolemia. Atheroscler. Suppl..

[190] Wong E., Goldberg T. (2014). Mipomersen (Kynamro): A Novel Antisense Oligonucleotide Inhibitor for the Management of Homozygous Familial Hypercholesterolemia. Pharm. Ther..

[191] Stefanutti C., Thompson G.R. (2015). Lipoprotein Apheresis in the Management of Familial Hypercholesterolaemia: Historical Perspective and Recent Advances. Curr. Atheroscler. Rep..

[192] Schwartz J., Padmanabhan A., Aqui N., Balogun R.A., Connelly-Smith L., Delaney M., Dunbar N.M., Witt V., Wu Y., Shaz B.H. (2016). Guidelines on the Use of Therapeutic Apheresis in Clinical Practice-Evidence-Based Approach from the Writing Committee of the American Society for Apheresis: The Seventh Special Issue. J. Clin. Apher..

[193] Connelly-Smith L., Alquist C.R., Aqui N.A., Hofmann J.C., Klingel R., Onwuemene O.A., Patriquin C.J., Pham H.P., Sanchez A.P., Schneiderman J. (2023). Guidelines on the Use of Therapeutic Apheresis in Clinical Practice—Evidence-Based Approach from the Writing Committee of the American Society for Apheresis: The Ninth Special Issue. J. Clin. Apher..

[194] Nugent A.K., Gray J.V., Gorby L.K., Moriarty P.M. (2020). Lipoprotein Apheresis: First FDA Indicated Treatment for Elevated Lipoprotein(a). J. Clin. Cardiol..

[195] Russi G. (2015). Severe Dyslipidemia in Pregnancy: The Role of Therapeutic Apheresis. Transfus. Apher. Sci..

[196] Shapero K., Countouris M., Chibisov I., Jeyabalan A., Berlacher K. (2023). Peripartum Lipid Apheresis: Novel Management of Familial Hyperlipidemia in Pregnancy. JACC Case Rep..

[197] Ogura M., Makino H., Kamiya C., Yoshimatsu J., Soran H., Eatough R., Perrone G., Harada-Shiba M., Stefanutti C. (2016). Lipoprotein Apheresis Is Essential for Managing Pregnancies in Patients with Homozygous Familial Hypercholesterolemia: Seven Case Series and Discussion. Atherosclerosis.

[198] Stefanutti C., Julius U., Watts G.F., Harada-Shiba M., Cossu M., Schettler V.J., De Silvestro G., Soran H., Van Lennep J.R., Pisciotta L. (2017). Toward an International Consensus-Integrating Lipoprotein Apheresis and New Lipid-Lowering Drugs. J. Clin. Lipidol..

[199] Reijman M.D., Kusters D.M., Groothoff J.W., Arbeiter K., Dann E.J., de Boer L.M., de Ferranti S.D., Gallo A., Greber-Platzer S., Hartz J. (2024). Clinical Practice Recommendations on Lipoprotein Apheresis for Children with Homozygous Familial Hypercholesterolaemia: An Expert Consensus Statement from ERKNet and ESPN. Atherosclerosis.

[200] Gupta M., Liti B., Barrett C., Thompson P.D., Fernandez A.B. (2022). Prevention and Management of Hypertriglyceridemia-Induced Acute Pancreatitis During Pregnancy: A Systematic Review. Am. J. Med..

[201] Dittrich E., Schmaldienst S., Langer M., Jansen M., Hörl W.H., Derfler K. (2002). Immunoadsorption and Plasma Exchange in Pregnancy. Kidney Blood Press. Res..

[202] Lindkvist B., Appelros S., Regnér S., Manjer J. (2012). A Prospective Cohort Study on Risk of Acute Pancreatitis Related to Serum Triglycerides, Cholesterol and Fasting Glucose. Pancreatology.

[203] Preiss D., Tikkanen M.J., Welsh P., Ford I., Lovato L.C., Elam M.B., LaRosa J.C., DeMicco D.A., Colhoun H.M., Goldenberg I. (2012). Lipid-Modifying Therapies and Risk of Pancreatitis: A Meta-Analysis. JAMA.

[204] Toskes P.P. (1990). Hyperlipidemic Pancreatitis. Gastroenterol. Clin. N. Am..

[205] Berglund L., Brunzell J.D., Goldberg A.C., Goldberg I.J., Sacks F., Murad M.H., Stalenhoef A.F.H. (2012). Evaluation and Treatment of Hypertriglyceridemia: An Endocrine Society Clinical Practice Guideline. J. Clin. Endocrinol. Metab..

[206] Vallejo-Vaz A.J., Fayyad R., Matthijs Boekholdt S., Kees Hovingh G., Kastelein J.J., Melamed S., Barter P., Waters D.D., Ray K.K. (2018). Triglyceride-Rich Lipoprotein Cholesterol and Risk of Cardiovascular Events Among Patients Receiving Statin Therapy in the TNT Trial. Circulation.

[207] Edison R.J., Muenke M. (2004). Mechanistic and Epidemiologic Considerations in the Evaluation of Adverse Birth Outcomes Following Gestational Exposure to Statins. Am. J. Med. Genet. A.

[208] Godfrey L.M., Erramouspe J., Cleveland K.W. (2012). Teratogenic Risk of Statins in Pregnancy. Ann. Pharmacother..

[209] Visseren F., Mach F., Smulders Y.M., Carballo D., Koskinas K.C., Bäck M., Benetos A., Biffi A., Boavida J.M., Capodanno D. (2021). 2021 ESC Guidelines on Cardiovascular Disease Prevention in Clinical Practice. Eur. Heart J..

[210] Poornima I.G., Pulipati V.P., Brinton E.A., Wild R.A. (2023). Update on Statin Use in Pregnancy. Am. J. Med..

[211] Costantine M.M., Cleary K. (2013). Pravastatin for the Prevention of Preeclampsia in High-Risk Pregnant Women. Obstet. Gynecol..

[212] Klevmoen M., Bogsrud M.P., Retterstøl K., Svilaas T., Vesterbekkmo E.K., Hovland A., Berge C., Roeters van Lennep J., Holven K.B. (2021). Loss of Statin Treatment Years during Pregnancy and Breastfeeding Periods in Women with Familial Hypercholesterolemia. Atherosclerosis.

[213] Holmsen S.T., Bakkebø T., Seferowicz M., Retterstøl K. (2017). Statins and Breastfeeding in Familial Hypercholesterolaemia. Tidsskr. Nor. Laegeforen.

[214] Laufs U., Parhofer K.G., Ginsberg H.N., Hegele R.A. (2020). Clinical Review on Triglycerides. Eur. Heart J..

[215] Brunzell J.D. (2007). Clinical Practice. Hypertriglyceridemia. N. Engl. J. Med..

[216] Magarian G.J., Lucas L.M., Colley C. (1991). Gemfibrozil-Induced Myopathy. Arch. Intern. Med..

[217] Ward N.C., Watts G.F., Eckel R.H. (2019). Response by Ward et al. to Letter Regarding Article, “Statin Toxicity: Mechanistic Insights and Clinical Implications”. Circ. Res..

[218] Miller D.B., Spence J.D. (1998). Clinical Pharmacokinetics of Fibric Acid Derivatives (Fibrates). Clin. Pharmacokinet..

[219] Pierce L.R., Wysowski D.K., Gross T.P. (1990). Myopathy and Rhabdomyolysis Associated With Lovastatin-Gemfibrozil Combination Therapy. JAMA.

[220] Jones P.H., Davidson M.H. (2005). Reporting Rate of Rhabdomyolysis with Fenofibrate + Statin versus Gemfibrozil + Any Statin. Am. J. Cardiol..

[221] Tsai E.C., Brown J.A., Veldee M.Y., Anderson G.J., Chait A., Brunzell J.D. (2004). Potential of Essential Fatty Acid Deficiency with Extremely Low Fat Diet in Lipoprotein Lipase Deficiency during Pregnancy: A Case Report. BMC Pregnancy Childbirth.

[222] Abu Musa A.A., Usta I.M., Rechdan J.B., Nassar A.H. (2006). Recurrent Hypertriglyceridemia-Induced Pancreatitis in Pregnancy: A Management Dilemma. Pancreas.

[223] Saadi H.F., Kurlander D.J., Erkins J.M., Hoogwerf B.J. (1999). Severe Hypertriglyceridemia and Acute Pancreatitis during Pregnancy: Treatment with Gemfibrozil. Endocr. Pract..

[224] Goldenberg I., Benderly M., Goldbourt U. (2008). Update on the use of fibrates: Focus on bezafibrate. Vasc Health Risk Manag..

[225] Bachmann C.M., Janitschke D., Lauer A.A., Erhardt T., Hartmann T., Grimm M.O.W., Grimm H.S. (2023). Gemfibrozil-Induced Intracellular Triglyceride Increase in SH-SY5Y, HEK and Calu-3 Cells. Int. J. Mol. Sci..

[226] Harris W.S., Connor W.E., Illingworth D.R., Rothrock D.W., Foster D.M. (1990). Effects of Fish Oil on VLDL Triglyceride Kinetics in Humans. J. Lipid Res..

[227] Simha V. (2020). Management of Hypertriglyceridemia. BMJ.

[228] Skulas-Ray A.C., Wilson P.W.F., Harris W.S., Brinton E.A., Kris-Etherton P.M., Richter C.K., Jacobson T.A., Engler M.B., Miller M., Robinson J.G. (2019). Omega-3 Fatty Acids for the Management of Hypertriglyceridemia: A Science Advisory From the American Heart Association. Circulation.

[229] Bays H.E., Ballantyne C.M., Kastelein J.J., Isaacsohn J.L., Braeckman R.A., Soni P.N. (2011). Eicosapentaenoic Acid Ethyl Ester (AMR101) Therapy in Patients with Very High Triglyceride Levels (from the Multi-Center, PlAcebo-Controlled, Randomized, Double-BlINd, 12-Week Study with an Open-Label Extension [MARINE] Trial). Am. J. Cardiol..

[230] Wierzbicki A.S., Kim E.J., Esan O., Ramachandran R. (2022). Hypertriglyceridaemia: An Update. J. Clin. Pathol..

[231] Nordgren T.M., Lyden E., Anderson-Berry A., Hanson C. (2017). Omega-3 Fatty Acid Intake of Pregnant Women and Women of Childbearing Age in the United States: Potential for Deficiency?. Nutrients.

[232] Emmett P.M., Jones L.R., Golding J. (2015). Pregnancy Diet and Associated Outcomes in the Avon Longitudinal Study of Parents and Children. Nutr. Rev..

[233] Middleton P., Gomersall J.C., Gould J.F., Shepherd E., Olsen S.F., Makrides M. (2018). Omega-3 Fatty Acid Addition during Pregnancy. Cochrane Database Syst. Rev..

[234] Olsen S.F., Secher N.J. (1990). A Possible Preventive Effect of Low-Dose Fish Oil on Early Delivery and Pre-Eclampsia: Indications from a 50-Year-Old Controlled Trial. Br. J. Nutr..

[235] Olsen S.F., Østerdal M.L., Salvig J.D., Weber T., Tabor A., Secher N.J. (2007). Duration of Pregnancy in Relation to Fish Oil Supplementation and Habitual Fish Intake: A Randomised Clinical Trial with Fish Oil. Eur. J. Clin. Nutr..

[236] Olsen S.F., Secher N.J. (2002). Low Consumption of Seafood in Early Pregnancy as a Risk Factor for Preterm Delivery: Prospective Cohort Study. BMJ.

[237] Yokoyama M., Origasa H., Matsuzaki M., Matsuzawa Y., Saito Y., Ishikawa Y., Oikawa S., Sasaki J., Hishida H., Itakura H. (2007). Effects of Eicosapentaenoic Acid on Major Coronary Events in Hypercholesterolaemic Patients (JELIS): A Randomised Open-Label, Blinded Endpoint Analysis. Lancet.

[238] Manson J.E., Cook N.R., Lee I.-M., Christen W., Bassuk S.S., Mora S., Gibson H., Albert C.M., Gordon D., Copeland T. (2019). Marine N-3 Fatty Acids and Prevention of Cardiovascular Disease and Cancer. N. Engl. J. Med..

[239] Bhatt D.L., Steg P.G., Miller M., Brinton E.A., Jacobson T.A., Ketchum S.B., Doyle R.T., Juliano R.A., Jiao L., Granowitz C. (2019). Cardiovascular Risk Reduction with Icosapent Ethyl for Hypertriglyceridemia. N. Engl. J. Med..

[240] Nakao J., Ohba T., Takaishi K., Katabuchi H. (2015). Omega-3 Fatty Acids for the Treatment of Hypertriglyceridemia during the Second Trimester. Nutrition.

[241] Goldie C., Taylor A.J., Nguyen P., McCoy C., Zhao X.Q., Preiss D. (2016). Niacin Therapy and the Risk of New-Onset Diabetes: A Meta-Analysis of Randomised Controlled Trials. Heart.

[242] Institute of Medicine (1998). Dietary Reference Intakes for Thiamin, Riboflavin, Niacin, Vitamin B6, Folate, Vitamin B12, Pantothenic Acid, Biotin, and Choline.

[243] Rader J.I., Calvert R.J., Hathcock J.N. (1992). Hepatic Toxicity of Unmodified and Time-Release Preparations of Niacin. Am. J. Med..

[244] Lewek J., Bielecka-Dabrowa A., Toth P.P., Banach M. (2024). Dyslipidaemia Management in Pregnant Patients: A 2024 Update. Eur. Heart J. Open.

[245] Crouse J.R. (1987). Hypertriglyceridemia: A Contraindication to the Use of Bile Acid Binding Resins. Am. J. Med..

[246] Tsuang W., Navaneethan U., Ruiz L., Palascak J.B., Gelrud A. (2009). Hypertriglyceridemic Pancreatitis: Presentation and Management. Am. J. Gastroenterol..

[247] Basar R., Uzum A.K., Canbaz B., Dogansen S.C., Kalayoglu-Besisik S., Altay-Dadin S., Aral F., Ozbey N.C. (2013). Therapeutic Apheresis for Severe Hypertriglyceridemia in Pregnancy. Arch. Gynecol. Obstet..

[248] Papadakis E.P., Sarigianni M., Mikhailidis D.P., Mamopoulos A., Karagiannis V. (2011). Acute Pancreatitis in Pregnancy: An Overview. Eur. J. Obstet. Gynecol. Reprod. Biol..

[249] Chen Z., Huang X., Zhang M., Han N., Ning Y. (2022). Rapid Reduction in Triglyceride Levels by Therapeutic Plasma Exchange in Patients with Hypertriglyceridemic Pancreatitis. J. Clin. Apher..

[250] Stefanutti C., Labbadia G., Morozzi C. (2013). Severe Hypertriglyceridemia-Related Acute Pancreatitis. Ther. Apher. Dial..

[251] Ewald N., Kloer H.U. (2012). Treatment Options for Severe Hypertriglyceridemia (SHTG): The Role of Apheresis. Clin. Res. Cardiol. Suppl..

[252] Syed H., Bilusic M., Rhondla C., Tavaria A. (2010). Plasmapheresis in the Treatment of Hypertriglyceridemia-Induced Pancreatitis: A Community Hospital’s Experience. J. Clin. Apher..

[253] Gavva C., Sarode R., Agrawal D., Burner J. (2016). Therapeutic Plasma Exchange for Hypertriglyceridemia Induced Pancreatitis: A Rapid and Practical Approach. Transfus. Apher. Sci..

[254] Kadikoylu G., Yavasoglu I., Bolaman Z. (2006). Plasma Exchange in Severe Hypertriglyceridemia a Clinical Study. Transfus. Apher. Sci..

[255] Lennertz A., Parhofer K.G., Samtleben W., Bosch T. (1999). Therapeutic Plasma Exchange in Patients with Chylomicronemia Syndrome Complicated by Acute Pancreatitis. Ther. Apher..

[256] Yeh J.H., Chen J.H., Chiu H.C. (2003). Plasmapheresis for Hyperlipidemic Pancreatitis. J. Clin. Apher..

[257] Kandemir A., Coşkun A., Yavaşoğlu İ., Bolaman Z., Ünübol M., Yaşa M.H., Kadıköylü G. (2018). Therapeutic Plasma Exchange for Hypertriglyceridemia Induced Acut Pancreatitis: The 33 Cases Experience from a Tertiary Reference Center in Turkey. Turk. J. Gastroenterol..

[258] Piolot A., Nadler F., Cavallero E., Coquard J.L., Jacotot B. (1996). Prevention of Recurrent Acute Pancreatitis in Patients with Severe Hypertriglyceridemia: Value of Regular Plasmapheresis. Pancreas.

[259] Wind M., Gaasbeek A.G.A., Oosten L.E.M., Rabelink T.J., van Lith J.M.M., Sueters M., Teng Y.K.O. (2021). Therapeutic Plasma Exchange in Pregnancy: A Literature Review. Eur. J. Obstet. Gynecol. Reprod. Biol..

[260] Perrone G., Brunelli R., Marcoccia E., Zannini I., Candelieri M., Gozzer M., Stefanutti C. (2016). Therapeutic Apheresis in Pregnancy: Three Differential Indications With Positive Maternal and Fetal Outcome. Ther. Apher. Dial..

[261] Perrone S., Brunelli R., Perrone G., Zannini I., Galoppi P., Di Giacomo S., Morozzi C., Pisciotta L., Stefanutti C. (2019). A Successful Term Pregnancy with Severe Hypertriglyceridaemia and Acute Pancreatitis. Clinical Management and Review of the Literature. Atheroscler. Suppl..

[262] Altun D., Eren G., Cukurova Z., Hergünsel O., Yasar L. (2012). An Alternative Treatment in Hypertriglyceridemia-Induced Acute Pancreatitis in Pregnancy: Plasmapheresis. J. Anaesthesiol. Clin. Pharmacol..

[263] Safi F., Toumeh A., Qadan M.A.A., Karaz R., AlAkdar B., Assaly R. (2014). Management of Familial Hypertriglyceridemia-Induced Pancreatitis during Pregnancy with Therapeutic Plasma Exchange: A Case Report and Review of Literature. Am. J. Ther..

[264] Rawla P., Sunkara T., Thandra K.C., Gaduputi V. (2018). Hypertriglyceridemia-Induced Pancreatitis: Updated Review of Current Treatment and Preventive Strategies. Clin. J. Gastroenterol..

